# Unravelling Convergent Signaling Mechanisms Underlying the Aging-Disease Nexus Using Computational Language Analysis

**DOI:** 10.3390/cimb47030189

**Published:** 2025-03-14

**Authors:** Marina Junyent, Haki Noori, Robin De Schepper, Shanna Frajdenberg, Razan Khalid Abdullah Hussen Elsaigh, Patricia H. McDonald, Derek Duckett, Stuart Maudsley

**Affiliations:** 1 Receptor Biology Lab., University of Antwerp, 2610 Wilrijk, Belgium; marinajf14@gmail.com (M.J.); haki.noori@student.kuleuven.be (H.N.); robin.deschepper@uantwerpen.be (R.D.S.); shanna.frajdenbrg@student.uantwerpen.be (S.F.); razan.khalidabdullahhussenelsaigh@student.uantwerpen.be (R.K.A.H.E.); 2 IMIM, Hospital del Mar Research Institute, 08003 Barcelona, Spain; 3 Department of Chemistry, KU Leuven, Oude Markt 13, 3000 Leuven, Belgium; 4 Lexicon Pharmaceuticals Inc., 2445 Technology Forest Blvd Fl 1, The Woodlands, TX 77381, USA; pmcdonald@lexpharma.com; 5 Department of Drug Discovery, H. Lee Moffitt Cancer Center, 12902 Magnolia Drive, Tampa, FL 33612, USA; derek.duckett@moffitt.org

**Keywords:** disease, aging, mechanisms, informatics, target, convergence, signal transduction, receptor, kinase, aging, stress

## Abstract

Multiple lines of evidence suggest that multiple pathological conditions and diseases that account for the majority of human mortality are driven by the molecular aging process. At the cellular level, aging can largely be conceptualized to comprise the progressive accumulation of molecular damage, leading to resultant cellular dysfunction. As many diseases, e.g., cancer, coronary heart disease, Chronic obstructive pulmonary disease, Type II diabetes mellitus, or chronic kidney disease, potentially share a common molecular etiology, then the identification of such mechanisms may represent an ideal locus to develop targeted prophylactic agents that can mitigate this disease-driving mechanism. Here, using the input of artificial intelligence systems to generate unbiased disease and aging mechanism profiles, we have aimed to identify key signaling mechanisms that may represent new disease-preventing signaling pathways that are ideal for the creation of disease-preventing chemical interventions. Using a combinatorial informatics approach, we have identified a potential critical mechanism involving the recently identified kinase, Dual specificity tyrosine-phosphorylation-regulated kinase 3 (DYRK3) and the epidermal growth factor receptor (EGFR) that may function as a regulator of the pathological transition of health into disease via the control of cellular fate in response to stressful insults.

## 1. Introduction

Human aging is a complex process that involves a panoply of coordinated molecular changes in cells and tissues throughout the body. These changes can include alterations in gene expression, protein structure and function, cellular signaling pathways, and the accumulation of several types of damage to biomolecules such as DNA, proteins, and lipids. This damage invariably causes a unidirectional diminution of physiological integrity that engenders loss of physiological functionality, resulting in an increased cellular vulnerability to morbidity and finally mortality [1,2,3,4,5,6,7]. With this deleterious effect of aging on cellular resilience—at a local and a systemic level—it is unsurprising that such a negative impact on the global cellular health status can lead to the increased incidence of multiple disease pathophysiologies including neurodegenerative conditions such as Alzheimer’s disease, osteoporosis, chronic kidney disease, schizophrenia, depression, and T2DM [8,9,10,11,12,13,14,15,16,17]. Given this significant intersection between the aging process and disease, it has recently been a subject of debate as to whether ‘pathological aging’ per se can be considered a specific disease [18,19]. As aging and the accumulation of damage is a natural process observed in nearly all organisms, it differs in a sense from aging-related diseases by its near universal presence in organisms, while aging-related diseases present at a much lower incidence rate [20,21,22]. Hence, the aging process itself is generally not considered to be a specific disease [23,24]. Aging, however, presents itself as a potent pathological process that facilitates, or perhaps even induces, the creation of disease(s) in elderly people [25] and can as such be considered a prime risk factor for the development of many of the world’s most debilitating disorders [26,27,28,29].

Given the profound linkage between aging and disease, it is ever more important to understand how this intersection may occur at the molecular level and which specific therapeutically tractable targets could be present at this nexus. Molecular aging is likely driven by a combination of both intrinsic and extrinsic factors that cause progressive changes that lead to imbalances in cellular homeostasis [30,31,32]. These factors can include genetic predisposition, environmental stressors such as radiation or oxidative stress, and lifestyle factors such as diet and exercise [33,34,35]. Some of the molecular changes, often codified as ‘hallmarks’ of aging include telomere shortening (leading to genomic instability), mitochondrial dysfunction, altered intercellular communication, oxidation-associated DNA damage, accumulation of senescent cells, and impaired protein quality control (proteostasis) [1,2]. These dysfunctions of normal cellular function invariably induce molecular damage—of which not all can be repaired [36]. This unrepaired damage thus accumulates over time, and this degree then results in the transition of healthy homeostasis to disrupted homeostasis—i.e., pathophysiology and disease [37,38].

In this context, it is evident that aging-related disorders share many common features: oxidative stress, DNA damage, metabolic disruption, and loss of cellular resilience [1,2,3,39,40,41], suggesting the potential for new multidimensional therapeutic strategies to mitigate this generic point of disease/aging genesis. Here, we have chosen to apply a multi-methodological approach to impartially identify tractable intervention mechanisms to control the key signaling features of the potential molecular intersection between cellular aging mechanisms and the ultimate disease process. To this end, we have used large language artificial intelligence models, interactomic analyses, semantic data mining, and molecular signature analysis to identify the signaling systems that could be manipulated to prevent aging-induced multi-disease etiology. Using this comprehensive combinatorial approach, we have identified a potentially new disease intervention mechanism linked to DYRK3 (Dual specificity tyrosine-phosphorylation-regulated kinase 3) and the epidermal growth factor receptor (EGFR).

## 2. Materials and Methods

### 2.1. Large Language Model-Based Data Curation

Using the free access large language model application ChatGPT v. 3.5 (https://chat.openai.com/: Open AI Inc., San Francisco, CA, USA), a simple semantic query was employed as follows: ‘Can you generate a list of 100 proteins associated with…’. This interrogation term was qualified with the insertion of a specific disease term: Coronary Heart Disease; Cancer; Chronic Obstructive Pulmonary Disorder; Stroke; Alzheimer’s Disease; Type II Diabetes Mellitus; Chronic Kidney Disease; Non-Alcoholic Fatty Liver Disease; Long-COVID; Major Depressive Disorder. The specific interrogator terms used for the diverse aging mechanisms were: genomic instability; telomere attrition; disrupted epigenetic regulation; disrupted proteostasis; disrupted nutrient sensing; mitochondrial dysfunction; disrupted cell-cell communication; cell senescence; cell frailty. To generate 100 protein lists of random proteins, the application Random Gene Set Generator (https://molbiotools.com/randomgenesetgenerator.php: molbioltools, Prague, Czech Republic) was employed. Random Gene Set Generator is a freely available web-based platform for the generation of random selections of genes from the genome of a specified organism. The random selection algorithm employed is based on the Mersenne Twister pseudorandom number generator (PRNG), and the source lists of genes have been parsed from species-specific genome annotation files that are readily available at Ensembl (https://useast.ensembl.org/info/data/ftp/index.html). For an alternative mechanism to generate text-associated protein lists, we employed the application PubPular v. 3.1.2 (https://heart.shinyapps.io/PubPular/: Denver, CO, USA). PubPular is an application that enables rapid bibliometric analysis, in conjunction with text mining and data curation, to generate prioritized proteins statistically associated with a specific input research topic [42]. All web-based applications were accessed on 1 July 2024.

### 2.2. Network Function Analysis

To generate and analyze protein-protein interaction (PPI) networks, STRING (https://string-db.org/) v. 12 was employed with the protein ID set as human STRING (Search Tool for the Retrieval of Interacting Genes/Proteins) [43]. It is a widely used database and analytical tool that integrates known and predicted protein-protein associations from multiple sources. STRING provides insights into how proteins interact within cellular processes, aiding in the understanding of biological functions and pathways. For clustering proteins into subgroups within a network, k means clustering was employed. Unless specifically stated otherwise, the minimum required interaction score level was set at 0.4. For multiplexed protein-protein/chemical network analysis, the application INDRA (Integrated Network and Dynamical Reasoning Assembler) and NDEX [44] were employed via the DarkKinome database system (https://darkkinome.org/) [45]. This database is a resource that focuses on the underexplored or ‘dark’ kinome—referring to kinases that are the subject of few studies compared to more commonly studied kinases. All web-based applications were accessed on 1 July 2024.

### 2.3. Pathway Enrichment Analysis

Multiple applications were employed to perform pathway enrichment analysis. Unless specifically stated, the primary mechanisms used was over representation analysis (ORA), using hypergeometric tests of probability. For all the mentioned pathway/Gene Ontology enrichment results stated, the basal inclusion criteria of a scored pathway/Gene Ontology term was the presence of at least two independent proteins enriching the specific category to a probability of at least *p* = 0.05. Two major multiplexed ORA analysis platforms were employed routinely in this study to facilitate specific pathway enrichment analyses, i.e., Enrichr (https://maayanlab.cloud/Enrichr/: [46,47,48]) and GeneTrail v.3.2 (https://genetrail.bioinf.uni-sb.de/: [49]). All web-based applications were accessed on 1 July 2024.

### 2.4. Data Representation and Venn Analyses

Word frequency analysis and word cloud generation was performed using the Word Frequency Counter from Code Beautify (https://codebeautify.org/word-frequency-counter) and Word Art (https://wordart.com/create). Venn diagram separation and analysis was performed using InteractiVenn [50] and the online Venn Diagram generator from the Biology and Evolutionary Genomics at Ghent University (https://bioinformatics.psb.ugent.be/webtools/Venn/). All web-based applications were accessed on 1 July 2024.

### 2.5. Cell Culture and Treatment

Human HEK293 cells (CRL 1573) were obtained from ECACC and cultured at 37 °C with 5% CO_2_ ambient tension, according to the approved culture protocols defined for these cells. Cells were maintained in Dulbecco’s Modified Eagle Medium (DMEM; Sigma-Aldrich, St. Louis, MO, USA) with 10% fetal bovine serum (FBS)-containing propagation media, supplemented with 1% Penicillin/Streptomycin antibiotics as previously described [51]. One day prior to experimentation, 3 × 10^6^ cells were seeded into 10 cm plates to obtain a 50–80% cell confluence on the day of the transfection. Cells were counted using a Luna II Automated Cell Counter (Invitrogen-Life Technologies, Thermo Fisher Scientific, Carlsbad, CA, USA). To induce oxidative stress, cells were treated with 100 nM hydrogen peroxide (H_2_O_2_/peroxide) for 90 min. Cellular proteins were extracted using an NP-40-based lysis buffer (150 mM NaCl, 50 mM Tris, 0.5% Sodium deoxycholate, 1% NP-40) supplemented with a phosphatase inhibitor cocktail (PhosSTOP, Roche Diagnostics, Rotkreuz, Switzerland) and a protease inhibitor cocktail (complete mini, Roche Diagnostics).

### 2.6. Immunoblot and Immunoprecipitation

Extracted proteins were separated on 4–12% SDS-PAGE (Life Technologies), transferred to PVDF membrane (Amersham, UK) and blocked using 5% BLOTTO milk. Primary antibodies for immunoblots: HER1/EGFR (sc-373746—Santa Cruz, CA, USA), DYRK3 (sc-390532—Santa Cruz), beta-Actin (A2228—Milipore Sigma, Sigma-Aldrich, St. Louis, MO, USA). The membrane was then incubated with species appropriate secondary antibodies conjugated to horseradish peroxidase (HRP), immune complexes were then identified using enhanced chemiluminescence (ECL, GE Healthcare, Chicago, IL, USA) and an Amersham imager 680 system. WB quantification was performed with GE-ImageQuant TL v.8 and Image J software v.1.54. Cerebral cortex lysates (1 mg/mL protein content), as described previously [52], were obtained from C57BL/6 mice, bred under NIH protocol numbers, 432-LCI-2015 and 433-LCI-2015, according to approval of the Institutional Review Board. All animal studies performed were approved according to the guidelines of the NIA Animal Care and Use Committee. EGFR/HER1 or DYRK3 were immunoprecipitated with 2 μg of either anti-EGFR or anti-DYRK3 antibody incubated with the clarified whole cell lysate in addition to 20 μL of a 30% slurry of protein G plus/protein A-agarose (Pierce). Following overnight incubation with agitation at 4 °C, immune complexes were collected by centrifugation (15 min, 14,000 rpm) and washed twice in ice-cold NP-40 based lysis buffer. Proteins were then removed from the immune complexes using an equal volume of 2× Laemmli sample buffer [51]. Immunoprecipitated proteins were then resolved via SDS-PAGE as previously described before selective immunoblotting.

### 2.7. Statistical Analyses

In each histogram or figure, the data are represented as the means ± SEM (standard error of the mean). Statistical analyses (Student’s *t*-test) were performed using GraphPad Prism version 9.5 (GraphPad Software, San Diego, CA, USA). Significance level is indicated in each figure as * *p* ≤ 0.05; ** *p* ≤ 0.01; *** *p* ≤ 0.001.

## 3. Results

### 3.1. Signature Generation for Common Diseases and Generic Aging Mechanisms

**Figure 1:**
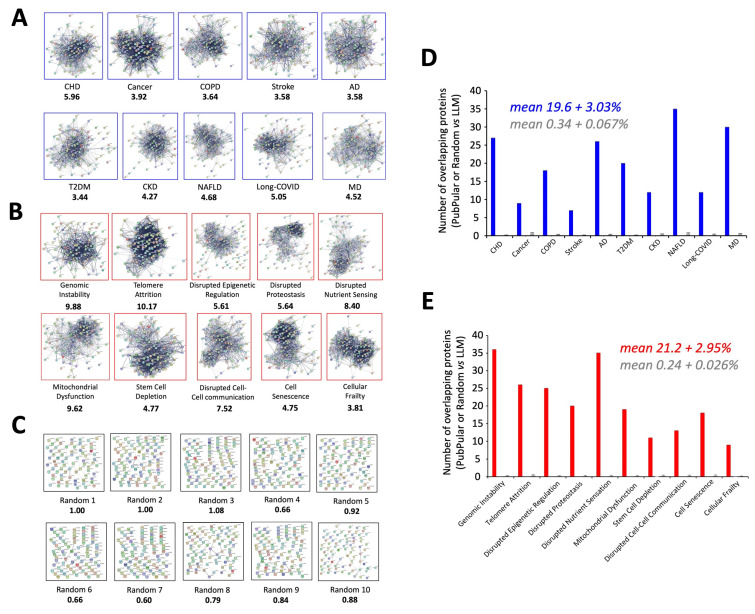
(**A**) Protein-protein interaction networks for commonly occurring disease processes. The 100 proteins identified in association with the defined diseases (coronary heart disease (CHD); cancer; chronic obstructive pulmonary disease (COPD); stroke; Alzheimer’s disease (AD); Type II diabetes mellitus (T2DM); chronic kidney disease (CKD); Non-alcoholic fatty liver disease (NAFLD); Long-Covid (also known as post-acute sequelae of SARS-CoV-2 infection (PASC)); major depression (MD)) were clustered into protein-protein interaction (PPI) networks using STRING. For all of the generated networks, the PPI enrichment probability was stated as *p* < 1.0 × 10^−16^. The values for the STRING-derived edge enrichment ratio (observed number of PPI network edges/expected number of PPI network edges) are depicted in the figure below the specific disease networks. (**B**) Protein-protein interaction networks for well-characterized aging pathomechanisms. The 100 proteins identified in association with the defined aging pathomechanisms: genomic instability; telomere attrition; disrupted epigenetic regulation; disrupted proteostasis; disrupted nutrient sensing; mitochondrial dysfunction; stem cell depletion; disrupted cell-cell communication; cell senescence; cellular frailty, were clustered into protein-protein interaction (PPI) networks using STRING. For all of the generated networks, the PPI enrichment probability was stated as *p* < 1.0 × 10^−16^. The values for the STRING-derived edge enrichment ratio (observed number of PPI network edges/expected number of PPI network edges) are depicted in the figure below the specific disease networks. (**C**) Protein-protein interaction networks for random protein lists. Hundred randomly selected proteins were clustered into protein-protein interaction (PPI) networks using STRING. These datasets were denoted Random 1–10. The PPI enrichment probability (*p*) for each of the random networks was as follows: Random 1—0.484; Random 2—0.58; Random 3—0.442; Random 4—0.895; Random 5—0.623; Random 6—0.892; Random 7—0.915; Random 8—0.16; Random 9—0.739; Random 10—0.32. The values for the STRING-derived edge enrichment ratio (observed number of PPI network edges/expected number of PPI network edges) are depicted in the figure below the specific disease networks. (**D**) Venn diagram set comparison of the disease specific datasets generated using PubPular and the LLM. The number of overlapping proteins found in specific diseases definitions (100 proteins each) from PubPular or the LLM. In addition, the overlap level of proteins between the LLM and randomly generated proteins lists is indicated. The mean percentage overlap of protein identities between the PubPular disease definition and the LLM definition was 19.6 + 3.03% while the random protein-LLM overlap mean was 0.34 + 0.067%). (**E**) Similar to the disease terms, the level of protein overlaps between PubPular definition of disease pathomechanisms and the LLM definitions is shown, compared to overlap with randomly generated 100 protein datasets. The mean percentage overlap of protein identities between the PubPular pathomechanism definition and the LLM definition was 21.2 + 2.95% while the random protein-LLM overlap mean was 0.24 + 0.026%.

We employed an openly available large language model (LLM) AI application (ChatGPT3.5: https://chat.openai.com/ accessed on 20 February 2025) to generate a nuanced and impartial assessment of the molecular protein associations with either a common disease or a generic aging mechanism. ChatGPT is an advanced conversational AI system based on a large language model (LLM) that uses deep learning techniques, specifically the transformer architecture, to understand and generate human-like semantic responses to query questions. This molecular signature generation using this LLM application was limited to a simple 100 protein signature for all the desired categories. Hence, the disease signatures were generated for: coronary heart disease (CHD); cancer; chronic obstructive pulmonary disease (COPD); stroke; Alzheimer’s disease (AD); Type II diabetes mellitus (T2DM); chronic kidney disease (CKD); Non-alcoholic fatty liver disease (NAFLD); Long-Covid (also known as post-acute sequelae of SARS-CoV-2 infection (PASC)); major depression (MD) (Table S1). At a similar scale (i.e., 100 proteins), similar signatures were made using the LLM for multiple aspects of the molecular aging process: genomic instability; telomere attrition; disrupted epigenetic regulation; disrupted proteostasis; disrupted nutrient sensing; mitochondrial dysfunction; stem cell depletion; disrupted cell-cell communication; cell senescence; cellular frailty (Table S2). To control for the LLM-based signature generation bias, similar magnitude (100 proteins) random protein lists were also created using the Random Geneset Generator from molbioltools (https://molbiotools.com/randomgenesetgenerator.php accessed on 20 February 2025). The specific 100 protein signatures were then analyzed for functional associations between the proteins using STRING-based analysis. The specific disease-based networks (Figure 1A) were assessed for their network edge enrichment (i.e., number of observed edges/numbers of expected edges), average node degree, and average clustering coefficient. A similar level of network investigation was applied for the mechanisms of aging (Figure 1B) and the ten random 100 protein datasets. It was evident that for both human diseases and aging mechanisms, there were significant differences in the network parameters (edge enrichment, average node degree, average clustering coefficient) compared to those generated for the random 100 protein networks (Figure 1C: Supplementary Figure S1).

To assess the specificity of the employed LLM to identify meaningful simple insights into complex processes, we compared it to the retrieval capacity of PubPular v3.1 (https://heart.shinyapps.io/PubPular/ accessed on 20 February 2025 [42]) that generates a semantics-based capacity to retrieve protein identities linked to an input search topic or disease identifier. We compared the retrieval capacity of PubPular (Table S3—Disease; Table S4—Pathomechanisms) to the LLM and then also to a similarly sized randomly generated dataset using simple overlapping protein analysis (Figure 1). Here, we found that for human diseases across all ten studied terms, there was a 19.6 + 3.03% overlap between PubPular retrieval and the LLM, compared to 0.34 + 0.067% overlap with the random data lists (comparing across all ten diseases this distinction was *p* < 0.0001, two-tailed *t*-test). A similar definite correlation between PubPular and LLM results was observed for the aging mechanisms: 21.2 + 2.95% overlap with LLM for PubPular retrieval versus 0.24 + 0.026% overlap with LLM for the random data (*p* < 0.0001 for cross-mechanism means). Hence, there is a strong and meaningful connection between the LLM and PubPular signatures despite their differential mechanistic construction.

**Figure 2:**
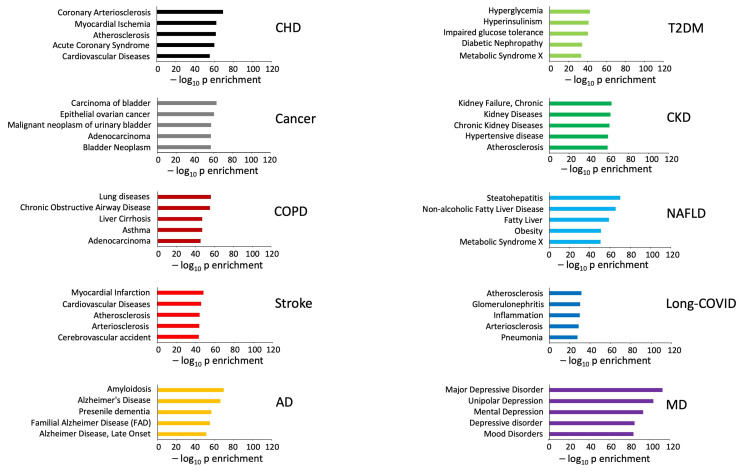
DisGeNET disease pathway enrichment analysis of the LLM-generated disease definitions. Using the DisGeNET curated databases, an enrichment analysis was performed for each of the specific 100 protein disease lists. Each panel denotes the top 5 most significantly enriched unbiased disease definitions. The probability scores are denoted on each panel as negative log10 transforms of the enrichment probability.

**Figure 3:**
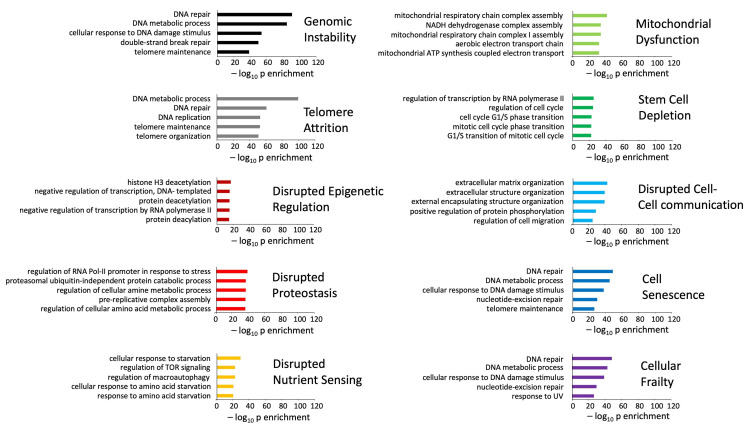
Gene Otology biological process enrichment analysis of the LLM-generated aging pathomechanisms. Using the Gene Ontology—Biological Process (GO-BP)—curated database enrichment analysis was performed for each of the specific 100 protein disease lists. Each panel denotes the top 5 most significantly enriched unbiased enriched GO-BP terms. The probability scores are denoted on each panel as negative log10 transforms of the enrichment probability.

The functional phenotypes of the human disease and aging mechanisms signatures were assessed using DisGeNET human disease pathway enrichment (https://www.disgenet.org/ accessed on 20 February 2025) [53] and Gene Ontology-Biological Process annotation (https://www.geneontology.org/ accessed on 20 February 2025) using the Enrichr analytical platform. Enrichr is an open access web-based platform designed for gene set enrichment analysis, and it integrates a wide variety of biological datasets. Given the innovative data collection approach, i.e., open source LLM, the resulting disease-based analytical outputs (DisGeNET) were highly accurate in identifying the specific disease phenotype from each LLM signature (Figure 2). The accuracy of the specific top 5 DisGeNET annotations indicates the high quality of the data retrieval from the LLM. A similar data accuracy was found with respect to the Gene Ontology-Biological Process annotation of the aging mechanism signatures (Figure 3). In this regard, the application of a simple LLM prompt has demonstrated a novel method for impartial data retrieval and curation.

### 3.2. Signature Refinement and Analysis for Core Properties

**Figure 4:**
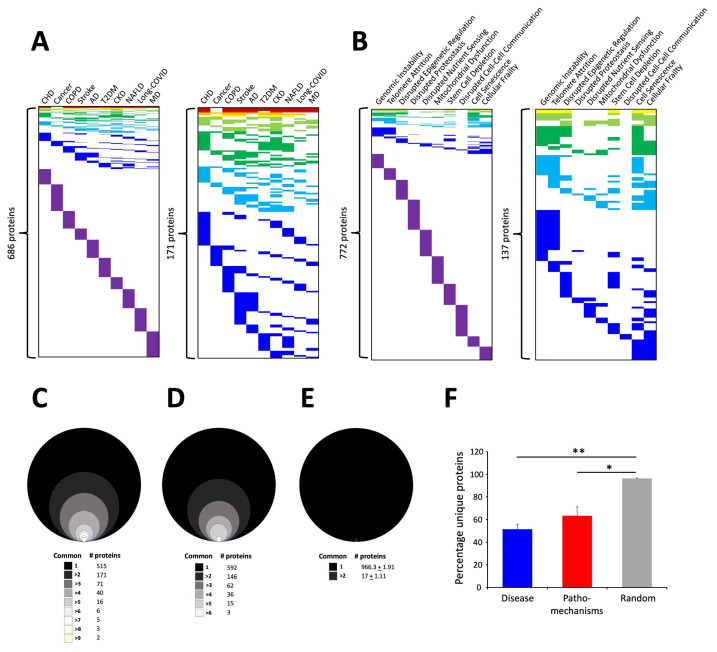
Protein distribution patterns for disease or pathomechanisms defined by the LLM. (**A**) The heatmaps depict the degree of commonality of specific proteins across the diverse disease processes. In the panel, the leftmost heatmap includes all retrieved proteins while the rightmost panel in (**A**) depicts the cross-disease distribution of proteins found in at least two different disease definitions. (**B**) As with panel (**A**), the heatmap on the left of the panel shows the total distribution of proteins across different pathomechanisms, while the heatmap on the right depicts the cross-pathomechanism distribution of proteins found in at least two different pathomechanism definitions. (**C**) Numerical distribution pattern of proteins across the ten LLM disease definitions. The diagram indicates the relative number of proteins that are found common with differing numbers of disease signatures. (**D**) Numerical distribution pattern of proteins across the ten LLM pathomechanisms definitions. As with panel (**C**), the relative number of proteins that are found common with differing numbers of pathomechanism signatures are shown. (**E**) Creating ten individual 100 identity protein lists the degree of commonality of proteins found with random data is shown in panel E in a comparable manner to panels (**C**,**D**). (**F**) The percentage of unique proteins found in either the disease, pathomechanisms, or random signatures is shown. Histogram-based data represent the means ± SEM (standard error of the mean). The significance level is indicated in each figure as * *p* ≤ 0.05; ** *p* ≤ 0.01.

Next, we assessed the degree of distinction of the human disease and aging mechanism protein signatures. Clustering the proteins in the two signature categories based on their commonality across the multiple diseases (Figure 4A) or aging mechanisms (Figure 4B) revealed that there was a greater overall level of commonality for proteins across the multiple disease (686 total distinct proteins) states compared to the different aging mechanisms (772 total distinct proteins). Given that the input in each of the categories (disease or mechanism) was 100 protein items each, it is interesting to note that at the specific protein level, the characteristic aging mechanisms appear to be more diverse at the molecular level compared to the diseases (Figure 4C,D). This suggests that disease signatures therefore represent some form of condensed biology of the aging process and that the etiological mechanism of inducing disease is more varied than the ultimate pathological processes causing disease. We further assessed the protein identity distribution using ten random 100 protein identity lists to compare to the disease or pathomechanisms protein distributions (Figure 4E). Comparing the specific datasets with the random datasets, it was evident that there was a clear distinction between specific retrieval and random retrieval processes. Assessing the percentage of proteins that were unique to a specific disease process or aging mechanism, we found that, on average across all ten items, the mean percentage of disease-associated proteins that were unique to a specific disease was 51.5 + 4.4%, while in contrast, 63.5 + 7.8% of the aging mechanism proteins were unique to a specific aging mechanism. The difference between these was non-significant but indicates the greater convergence of disease proteins compared to aging mechanism driving proteins (Figure 4E). However, when compared to the percentage of list-specific proteins found in the random distribution diagram, both disease and pathomechanisms demonstrated a significant difference (Figure 4E). This would suggest that a greater level of diversity in the transition between health and disease exists in the damage-related mechanisms, and once a specific ‘tipping point’ of damage is reached, then aging-related disease progress occurs in a relatively generic manner.

### 3.3. Multilevel Functional Analyses of Aging Mechanisms and Disease Processes

**Figure 5:**
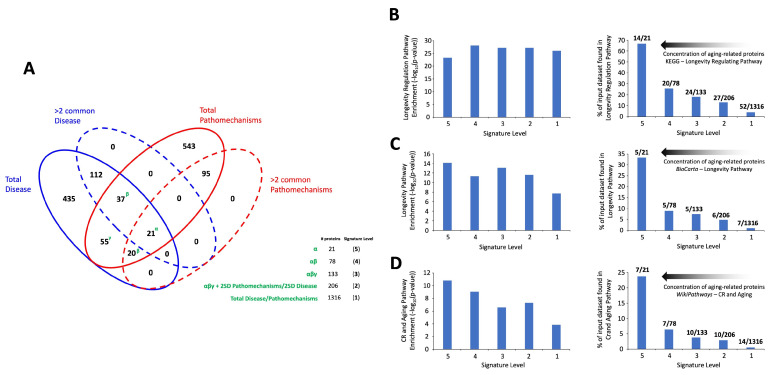
Venn diagram separation of the disease or pathomechanism LLM datasets. (**A**) Four-way Venn diagram separation of the proteins comprising total disease (solid blue line), >2 commonality disease proteins (dashed blue line); total pathomechanisms (solid red line), and >2 commonality pathomechanism datasets. To create distinct levels of data analysis the datasets outlined in the panel were constructed as follows. Level 5 = 21 proteins common to all four of the initial input datasets (totals and >2 commonalities across ten diseases/mechanisms). Level 4 = 78 proteins (Level 5 plus the additional 57 proteins found in at least three out of four of these datasets). Level 3 = 133 proteins (Level 5 and 4 plus the additional 55 proteins found common between the disease and mechanisms datasets that were only found in one category). Level 2 = 206 proteins (Level 5, 4, 3 plus additional 73 proteins found in the datasets of either disease or mechanisms demonstrating at least three category commonalities (>2 SD away from the total disease/mechanism population mean). Level 1 = 1316 proteins (comprises the total of the LLM-generated data. (**B**) KEGG pathway ‘Longevity Regulating Pathway’ enrichment analysis of Level 1 to 5 data (left panel). At each of the analytical levels for the ‘Longevity Regulating Pathway’ enrichment, the percentage of the total input dataset that was used for the pathway enrichment that belonged to this specific pathway was calculated. For each histogram bar, the number of input proteins that populated this pathway (Longevity Regulating Pathway) is denoted next to the total input protein dataset size (i.e., 14 proteins found in the pathway from the total of 21 input proteins). Panels (**C**,**D**) depict similar data for the specific enrichment of the BioCarta Longevity Pathway (**C**) and the WikiPathways CR and Aging pathway (**D**). For all three panels, there is a clear increase in the percentage of the input dataset used that falls within the specific aging-associated pathway from Level 1 ascending to Level 5.

Next, we generated a multilevel approach to investigate the potential mechanistic convergence of aging-related mechanisms and disease processes. To this end, we created a cohort of datasets extracted from the LLM-derived signatures. As we have previously described, there are two initial levels of interrogation that are apparent with respect to analysis across the complete range of aging-related diseases or aging mechanisms, i.e., proteins that were unique to just one disease/mechanism or ones that were shared. Using this simple parsing mechanism, we created a Venn diagram using the whole disease or mechanism datasets overlapping with the datasets exclusively for those proteins shared across at least two diseases or mechanisms (Figure 5). With this simple four-way (total disease; >2 protein common disease; total mechanisms; >2 protein common mechanisms) overlap analysis, we found that there were 21 proteins completely common to all the four datasets. In addition to this core of 21 proteins, there were 57 additional proteins that were found in at least three of the four distinct datasets (37 proteins common to total diseases, >2 protein common disease, total mechanisms; 20 proteins common to total disease, >2 protein common mechanisms, total mechanisms). The final group of proteins that displayed some level of commonality between the aging related diseases and mechanisms were 55 proteins (found only once in either a specific disease or mechanism) common only to the total disease or mechanisms datasets.

In addition to the simple parsing process of whether disease or mechanism proteins are found in more than one respective disease or mechanism category, we applied a total protein analysis of the total protein cohort in each case and assessed what level of commonality across either diseases or mechanisms was greater than two standard deviations above the mean number of disease or mechanism commonalities. Hence, across the ten diseases, we found that there were 71 proteins that were found in at least three different diseases—with the mean number of commonalities across all the disease-associated proteins being 1.45 + 2SD of 2.065. As the cut-off would fall somewhere between 3 and 4 commonalities, we employed the 71 detailed proteins that possessed at least three different disease commonalities for further investigation. Applying this metric to the mechanisms datasets, we identified 55 proteins that possessed at least three mechanism commonalities (mean commonalities plus 2SD deviation was 1.29 + 2 SD of 1.51).

We next combined these two metrics approaches to generate five distinct levels of dataset investigation (Table S5). Hence, we constructed five distinct levels of datasets to intensify the degree of convergence between aging-related disease and aging drive mechanisms. Level 5 comprises the 21 proteins common to all four of the initial input datasets (totals and >2 commonalities across ten diseases/mechanisms). Level 4 includes this cohort of proteins plus the additional 57 proteins found in at least three out of four of these datasets (generating a total of 78 proteins). Level 3 (133 proteins) comprises these 78 proteins plus the additional 55 proteins found common between the disease and mechanisms datasets that were only found in one category. Level 2 (206 proteins) was generated by supplementing the Level 3 list with additional proteins that were found in the datasets of either disease or mechanisms demonstrating at least three category commonalities (>2 SD away from the total disease/mechanism population mean). Level 1 comprises the total of the LLM-generated data, i.e., 1316. Proteins (Figure 5A).

**Figure 6:**
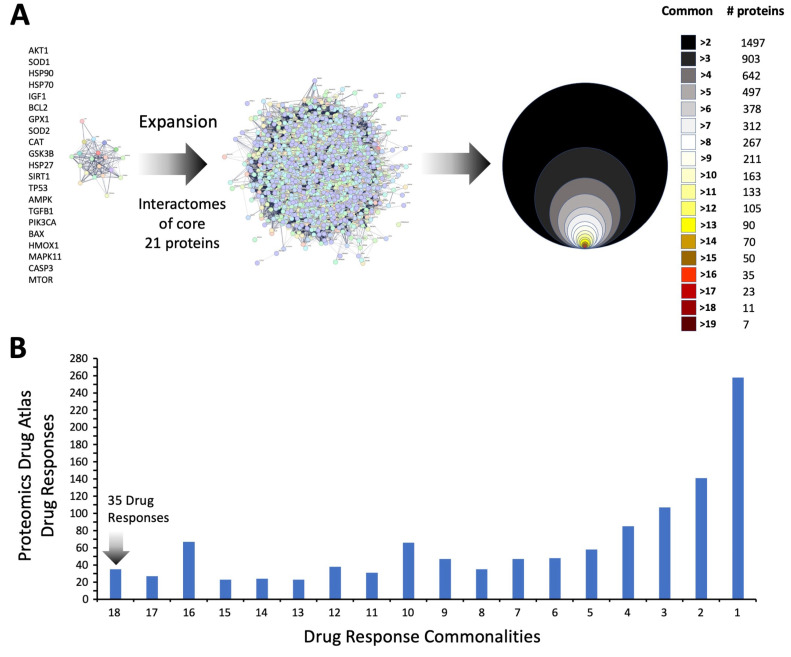
Interactome-based expansion of the Level 1 disease-pathomechanism core dataset. (**A**) An expansion dataset of proteins found to be associated (in at least seven different curated protein-protein association databases) with the 21 individual Level 1 proteins were created. The protein distribution pattern, i.e., how often a specific protein is found to be common between the original 21 protein lists, is depicted. In total, 1497 Level 1 core interacting proteins were found. At the most consistently found level there were 7 proteins found in 19 out of 21 input dataset lists. (**B**) By Generating Proteomics Drug Atlas Drug Response enrichment analysis profiles to the different protein commonality datasets (excluding the >19 common protein lists as no significant enrichment was found), we found 35 Proteomics Drug Atlas Drug Responses that were common to all the input dataset analysis streams.

Applying classical Over Representation Analysis (ORA) over pathway analysis of the five different data levels, we found a strong representation of aging-associated biology across all the five data levels (Figure 5). For several distinct forms of signaling pathway analysis (KEGG Pathway Analysis—Figure 5B; BioCarta—Figure 5C; WikiPathways—Figure 5D), a highly significant enrichment of KEGG—‘Longevity Regulating Pathway’, BioCarta—‘Longevity Pathway’, and WikiPathways ‘Calorie Restriction (CR), and Aging’ was found (Figure 5B–D). In general, the degree of enrichment probability was relatively similar for these specific signaling pathways from Level 1 to Level 5 of the different scale datasets (Figure 5B–D—left panel). Interestingly, in contrast to this relative consistency of enrichment, it was clear that the percentage of proteins in these aging-associated signaling pathways decreased significantly from level 5 to Level 1 (Figure 5B–D—right panel). Hence, a profound concentration of the aging-specific proteins was found in the Level 5 dataset compared to the other data levels. This suggests that with respect to common disease-generating processes, the 21 proteins comprising this level (Level 5) of data representation may have a profound impact on the progress and etiology of aging-associated disease. While it is clear that this core of proteins may be pivotal in the aging-disease nexus, we next performed an informatic expansion process. This involved the identification of the associated protein interactomes for each of these 21 ‘Level 5’ proteins using seven different protein-protein association databases: BioGRID (https://thebiogrid.org/) [54]; The Molecular INTeraction Database—MINT (https://mint.bio.uniroma2.it/) [55]; STRING (https://string-db.org/); GeneShot (https://maayanlab.cloud/geneshot/) [56]; PubPular [42] (https://heart.shinyapps.io/PubPular/); IntACT Molecular Interaction Database—IntACT (https://www.ebi.ac.uk/intact/home) [57]; Humanbase (https://hb.flatironinstitute.org/) [58]. Employing these diverse curated databases, protein-protein interaction (PPI) datasets for each of the 21-core aging-disease proteins were created. Across the seven curated databases, the average total proteins for the 21 core proteins were 1890 + 121.25. To refine this extraction process, interacting proteins that were identified in at least two proteins between the seven PPI databases were also assessed with an average of 399 + 37.64 proteins found in at least two databases. These proteins, found in at least two of the seven PPI databases for each protein were then aggregated to form the 21-protein expansion (Table S6). The distribution, across the different proteins from the core 21, of these proteins is described in Figure 6A. This network of proteins—all verified as protein associates to the original core 21 aging-disease proteins—likely represents an important potential cohort of proteins that constitutes an important target network for interdicting aging-driven disease. To further investigate this, we then interrogated this expansion network with the 2023 edition of the curated Proteomics Drug Atlas of therapeutic molecule signatures [59].

### 3.4. Therapeutic Signature Analysis of the Aging-Disease Nexus Core Expansion

**Figure 7:**
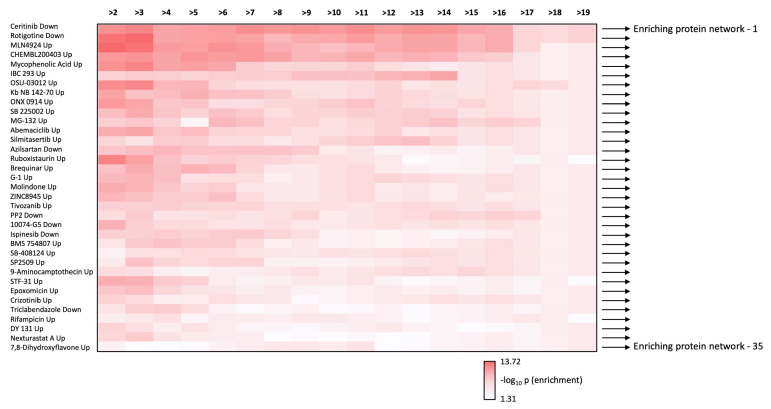
Heatmap for the common disease-pathomechanism enriched proteomic drug responses. The heatmap indicates the enrichment probabilities for all the completely common 35 drug responses. The associated key indicates the negative log10 transform the Proteomics Drug Atlas Drug Response enrichment probability score.

**Figure 8:**
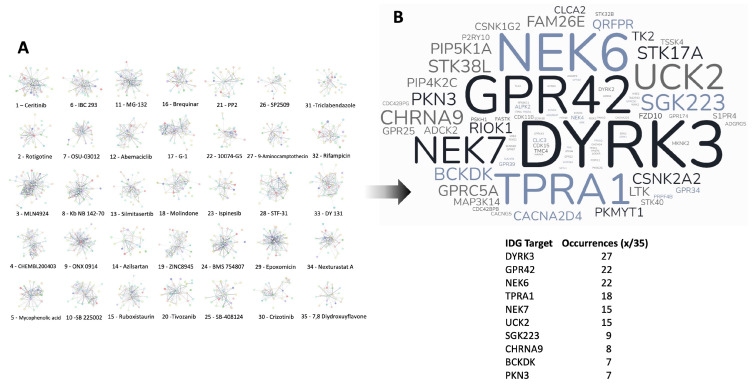
Frequency analysis of proteins linked to conserved Proteomics Drug Atlas Drug Response signature. (**A**) STRING-derived protein networks for the specific disease-pathomechanism proteins that significantly populate the conserved 35 drug response networks (1–35). The frequency of proteins found within all these 35 networks is depicted in panel (**B**) as a proportional word cloud. The kinase DYRK3 was found in 27 of the 35 drug response networks.

We next performed Proteomics Drug Atlas (PDA) ORA enrichment analysis of the multiple data levels, i.e., how many core-21 dataset expansion interactome commonalities (from >2 to >18 commonalities), of the expansion dataset. We found that there were 35 PDA therapeutic signatures that were found in all the expansion dataset series (Figure 6B). Hence, these PDA molecular signatures must share some important molecular features key to the regulation of the aging-disease nexus. We aggregated all the proteins identified in the core 21 expansion cohort that were enriched in the 35 common PDA therapeutic signatures (Figure 7 and Figure 8) and then analyzed the frequency of incorporation of specific proteins across these different 35 therapeutic signatures. We found the protein with the highest inclusion frequency, i.e., 27 times out of the 35 signatures was the Dual specificity tyrosine-phosphorylation-regulated kinase 3 (DYRK3).

DYRK3 is a member of the DYRK (dual-specificity tyrosine-regulated kinase) family of protein kinases—this comprises two Class I kinases (DYRK1A, DYRK1B) and three Class II kinases (DYRK2, 3, and 4) [60]. DYRK3 is a dual function kinase and is thus capable of phosphorylating both serine/threonine and tyrosine residues in substrate proteins. DYRK3 has been shown to be involved in multiple cellular processes that can affect aging/longevity including cell cycle regulation, DNA damage response, stress response, and protein stabilization [61,62,63]. Given these features, it is not surprising that an altered expression or dysregulation of DYRK3 has also been associated with certain cancers and neurodegenerative diseases [64,65,66].

We next investigated how the functional signature of DYRK3 may intersect with the aging-disease nexus core 21 expansion. First, we created an unbiased interactomic interpretation of human DYRK3 (UniProt ID: O43781) using a seven database (BioGRID, MINT, STRING, GeneShot, PubPular. IntACT, Humanbase) aggregation process. Using these diverse platforms, we accumulated a 321 PPI signature for DYRK3 (Figure 8) that we then used to identify the total number of DYRK3-associated factors in the expansion 1497 dataset. We thus found 73 DYRK3-expansion common proteins—then this intersection was repeated with 10 random datasets each comprising 321 randomly-chosen proteins and the level of random intersection with the expansion dataset was only 20.5 + 1.66 (mean + SEM). This random protein intersection process was assessed across all the various levels of protein commonalities for the expansion dataset. In each case, the actual number of DYRK3-associated factors was consistently above the level of random protein intersection (Figure 8).

### 3.5. Mechanistic Investigation of the DYRK3-Aging/Disease Nexus

**Figure 9:**
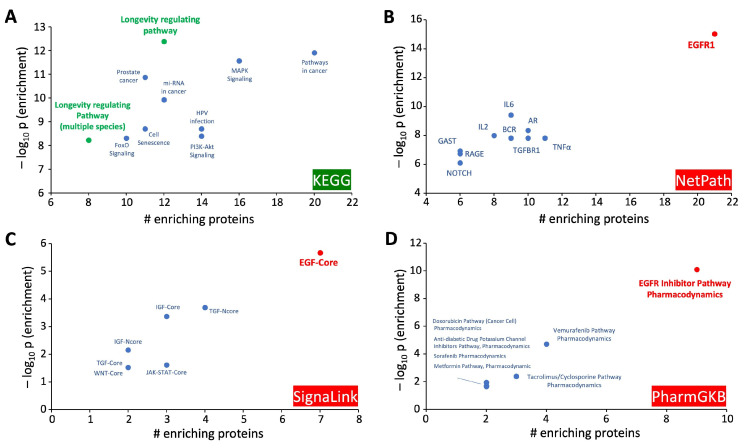
Multifaceted pathway enrichment analysis of the DYRK3-specific disease-pathomechanism dataset. Using the 73 DYRK3-specific proteins extracted from the >2 disease-pathomechanism nexus dataset, the following enrichment paradigms were performed: (**A**) KEGG pathway analysis; (**B**) NetPath analysis; (**C**) SignaLink pathway analysis; (**D**) PharmGKB pathway analysis. For each analytical output, the top 10 enriched pathways are indicated via a combined metric correlating the negative log10 transform of the enrichment probability value and the specific number of enriching proteins from the input DYRK3-associated dataset.

Given our identification of the potential therapeutic importance of DYRK3 in the aging/disease nexus, we decided to characterize the functionality of the intersection protein dataset between DYRK3 and the aging/disease nexus expansion. To this end, we found that—recapitulating our initial posit—that this dataset is indeed focused on regulating longevity (Figure 9) after KEGG pathway investigation of the 73 protein DYRK3-aging/disease intersection. With further pathway-based interrogation of this target dataset, we found a consistent presence of EGFR/EGF-associated connections for the novel dataset (Figure 9). To assess if this association could be verified via other metadata sources, we consulted the DarkKinome database [45]. Using the INDRA (Integrated Network and Dynamical Reasoning Assembler) platform, we identified the functional intersection between EGFR and DYRK3 [44]. INDRA is a computational platform that allows the construction, analysis, and simulation of biological networks based on the integration of information from various biological data sources. Further reinforcing the importance of DYRK3 in the aging process, the INDRA analysis revealed functional associations with multiple factors linked to aging-related disease therapy/pathophysiology (NEDD4L—[67]; SIRT1—[68]; mTORC1—[69]; CREB—[70]) (Figure 9).

**Figure 10:**
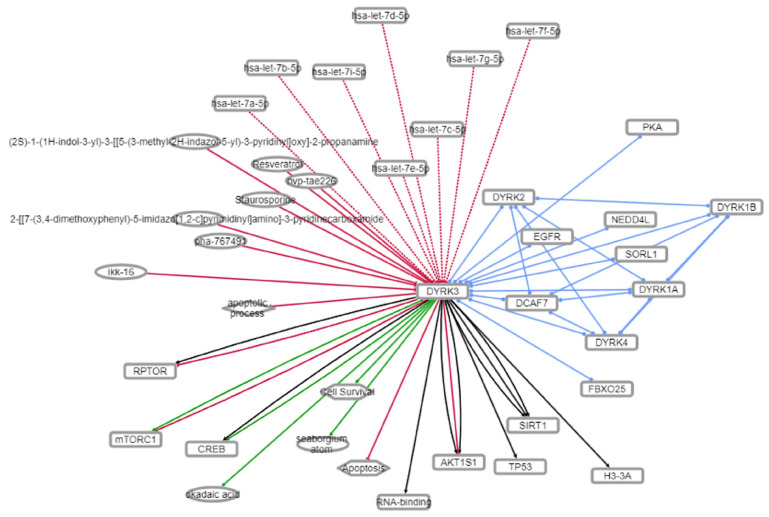
DarkKinome analysis of the functional sequelae of DYRK3. The knowledge graph depicted using NDEX v. 2.6. indicates the functional connections between the kinase DYRK3 and multiple forms of functional targets.

To develop a more in-depth inspection of the potential DYRK3-EGFR association, we created a PPI dataset (in a comparable manner to DYRK3) for EGFR and then assessed the degree (versus random datasets) of intersection between DYRK3 and EGFR (Figure 10).

**Figure 11:**
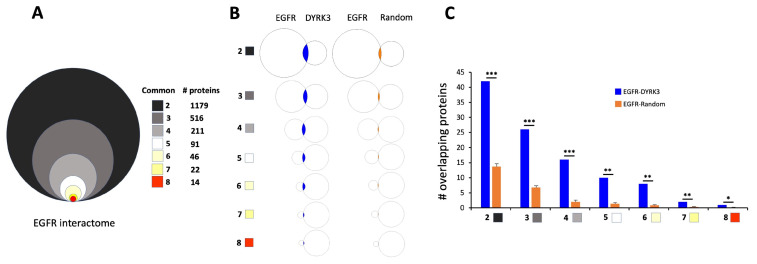
EGFR and DYRK3 share a profound functional interaction. (**A**) A functional EGFR interactome was created using eight independent protein interaction databases. The distribution of individual proteins across multiple databases is indicated in the color-coded proportional diagram. (**B**) Proportional Venn diagrams indicate the degrees of protein identity overlap between all levels of EGFR interactome protein commonalities with either the full DYRK3 interaction dataset (321 proteins) or the mean of 10 random (321 proteins) datasets. EGFR-DYRK3 intersections are denoted in blue, while the EGFR-Random intersections are denoted in orange. (**C**) Histogram representation of the degrees of protein overlap between the multiple levels of EGFR dataset interrogation with either the DYRK3 specific (blue bars) or the random (orange bars) datasets. Histogram-based data shown represent the means ± SEM (standard error of the mean). The significance level is indicated in each figure as * *p* ≤ 0.05; ** *p* ≤ 0.01; *** *p* ≤ 0.001.

Our assembled EGFR PPI dataset (created using multiple PPI databases) comprised a total of 5059 proteins with 1179 proteins found common between at least two PPI databases. This 1179 protein dataset and those protein groups with greater levels of PPI database commonality (i.e., >2, 3, 4, 5, 6, 7) were then assessed for their intersection (compared to random datasets) with the DYRK3 321 PPI dataset. At each of the EGFR-DYRK3 intersection levels, we found a highly significant association between EGFR and DYRK3 datasets—compared to random protein datasets of the same size as the DYRK3 dataset. When comparing the 1179 >2 commonality EGFR PPI dataset with the DYRK3 PPI dataset, we found 42 proteins that represent the functional link between these two factors. When we investigated (employing STRING network analysis) the synergistic functional relationship between EGFR-DYRK3, it was evident that a highly nuanced interconnection between EGFR functionality and aging/longevity mechanisms was present in the EGFR-DYRK3 nexus. Hence, multiple proteins within the EGFR-DYRK3 nexus were simultaneously involved in aging disease/mechanisms and EGFR activity. Expanding our analytical pipeline to this 42-protein intersection (Figure 11), we found that this intersection participated in the etiology of multiple disease processes (using Jensen Disease database: https://diseases.jensenlab.org/) that encompass many of the initial age-related disease factors targeted, i.e., hyperglycemia (T2DM), cancer, fatty liver disease (NAFLD), lung disease (COPD), and brain disease (AD, MDD, Stroke).

With respect to the potential effect on transcriptional effects, we observed the most intense enrichment probability for SNAI2, which controls (among many other functions) cell stemness in the context of aging/cancer/metabolic dysfunction [71,72,73]. Given the importance of the EGFR in both aging [74,75] and oncology [76], we assessed the potential EGFR-DYRK3 enrichment of oncogenic signatures using the MSigDB (Molecular Signatures Database: https://www.gsea-msigdb.org/gsea/msigdb/). Using the Oncogenic Signatures section of MSigDB, we found an interesting series of closely-associated enrichments, i.e., ones involving EGFR, BMI1 (Polycomb complex protein BMI-1), and MEL18 (aka Polycomb group RING finger protein 2—PCGF2). It is interesting to note that both BMI1 and MEL18 also appear to play a reciprocal role in controlling cell stemness and cell fate [77,78,79]. Using an additional subset of the MSigDB, i.e., the chemical and genetic perturbations (CGP), we found another highly enriched member of the Polycomb group RING finger protein 2 (PRC2) complex, i.e., SUZ12. It is interesting to note that healthy aging is associated with a close control of PRC2 complex activity [80,81,82]. We additionally found a specific enrichment of SUZ12 again using the EGFR-DYRK3 intersection data through the ESCAPE database (https://www.maayanlab.net/ESCAPE/) that curates evidence of stem cell pluripotency [83,84]. This suggests that the functional link between EGFR and DYRK3 may exert an important function in the aging-disease nexus via the control of cellular fate and stemness. This connection was further reinforced using TF Perturbations followed by expression (via Enrichr interrogation, of the NCBI Gene Expression Omnibus: https://www.ncbi.nlm.nih.gov/geo/) enrichment analysis. Using this platform, we found that the strongest enrichment was for PRRX1, which has been shown to regulate cell stemness in the setting of aging and oncology [85,86,87,88]. These data suggest that perhaps one of the most important anti-aging functions of the EGFR-DYRK3 relationship could be controlling age-associated damage and disease progression via the control of cellular fate in times of cell stress and damage. Therefore, targeting this network controlled by the EGFR-DYRK3 axis may represent an important target for the interdiction of multiple age-related diseases.

## 4. Discussion

Here, we have investigated the potential importance of a novel form of unbiased data curation for complex molecular biological situations, i.e., unravelling novel therapeutic mechanisms for interventions in aging-related disorders. Here, we have initially generated novel molecular signatures for both aging-related diseases as well as molecular drivers of the aging process at the cellular level. To this end, we employed ChatGPT 3.5 as a mechanism by which to identify protein cohorts linking aging diseases to aging mechanisms. Using a similar mechanism of molecular signature generation, i.e., queries applied at the same time using a standardized semantic input phrase that can generate an output of identical size.

Large language model artificial intelligence (AI) systems such as ChatGPT-3 have been making significant contributions to biomedical science in recent years. These open use applications have the potential to improve our understanding of complex biological systems and accelerate the development of new treatments for a wide range of diseases [89,90,91]. LLMs, in particular, and AI, in general, are especially useful with respect to the capacity to comprehensively appreciate the gestalt nature of large datasets such as genomics, proteomics, and medical imaging [14,15,92,93,94,95,96]. AI algorithms possess capacities to assimilate and analyze large ‘omic’-level datasets and thus identifying cryptic patterns and relationships that would be difficult or impossible for humans to detect. This has led to new insights into the underlying causes of diseases and hopefully the development of more effective treatments. Here, we have employed the capacity of an LLM to perform a novel aggregation of molecular signatures of highly complex processes, i.e., aging related diseases and molecular mechanisms of aging pathophysiology. Allowing a facile capacity to create these customized signatures adds an extra level of high-dimensionality data analysis that may indeed possess some benefits over traditional concept clustering [97,98,99,100]. For example, LLM base training sets can be rapidly updated and thus may include more novel insights compared to databases that may be modified at a lower frequency. In addition, these models can also employ a broader range dataset than classical informatic platforms. While LLM technologies bring novel aspects of database interrogation, it is vital that benchmarking and integration with other informatic systems still take place to ensure that sole reliance on LLMs is not pursued. It is important to note that LLMs can generate signature protein lists through condensing multiple text sources that will also be based on empirical research data and domain expert human curation. While providing a novel mechanism of text-based research, it is important to consider the potential limitations of LLM-based investigation. Training data bias is a common issue for LLMs and can depend on the relative surfeit or dearth of previous experimentation/text concerning a specific protein in signaling networks. Hence, data can be biased by trends in research concerning protein targets or disease burdens. The time to curate LLM databases can also be an issue with respect to inclusion of the most cutting-edge data into the training datasets. With respect to protein interaction/association databases, there is also the issue of whether the coincidence of protein expression in a specific dataset specifically guarantee functional interaction (either physically or indirectly). Associations based on the co-occurrence of proteins and diseases in the training data may not always align with experimentally validated findings. Hence, correlation does not always imply causation, so inferred protein-disease links may reflect shared experimental contexts rather than direct relevance. Appreciating the presence of these limitations of LLM systems, it is important to introduce mitigation strategies to address these issues. Hence, we cross-checked with other databases, demonstrated functional relevance of extracted data using empirical data investigation (e.g., INDRA, PDA), verified the quality of data with classical enrichment analyses, and have rigorously compared the veracity of the generated profiles using unbiased random data interrogation simultaneously. Here, we have demonstrated how a simple integration of LLM and classical informatic investigation can be used to create a novel mechanism for investigating complex biology and pathophysiology. Future development and refinement of LLMs alongside standard bioinformatic resources will likely improve the efficacy of this process to define disease mechanisms and therapeutic effects.

In this current research, the initial LLM-derived molecular signatures (Figure 1) were benchmarked using standard ORA-based pathway/Gene Ontology enrichment analysis (Figure 2). Thus, even with a distinct mode of generation, these signatures were effective condensations of the specific concept used to assemble the lists. From these signatures, we discovered a potential distinction between proteomic convergence between two different concepts in aging, i.e., the relative difference in molecular convergence between aging-related disease and molecular aging mechanisms. Thus, we found that there was a greater diversity of proteins involved in aging mechanisms whereas the proteins associated with age-related disease showed a stronger level of convergence (Figure 3). It has been shown in several conditions that across complex syndromic conditions, there are often observed convergences of protein complexity [101,102,103,104]. Using our combinatorial informatics approach, we were able to identify a core series of 21 protein factors (Figure 4) that were reliably found in a data scale-independent manner to link both aging-related disease and aging mechanisms. We found that this 21-protein cohort possessed a profound concentration of factors associated with longevity (Figure 5), suggesting that this data cohort represents a microcosm of the aging-disease nexus. These factors likely function as consistent drivers/regulators of the stressful responses to insults that cause aging (e.g., oxygen radical damage) as well as regulators of the eventual disease-inducing pathophysiological process. As this cohort may represent an extremely important therapeutic target, we performed an informatic ‘expansion’ process to capture as much as the functional profundity of this aging-nexus core (Figure 6). This ‘expansion’ created a rationally generated functional interactomic signature that could be employed for therapeutic screening analysis. We investigated therapeutic intervention enrichment in this core-21 expansion using the Proteomics Drug Atlas 2023 [59]. Across multiple conservance levels within the core-21 PPI expansion, we found a significant enrichment of 35 therapeutic signatures that were consistently found across all scale levels in the expansion (Figure 7). Upon inspection of the proteins involved with these 35 significantly enriched therapeutic interventions, we found that the most consistently represented signaling factor was the dual specificity kinase DYRK3 (Figure 8). With respect to its potential role in the aging-disease nexus, DYRK3 has been shown to control cell survival dynamics through its ability to phosphorylate the aging regulator SIRT1 [62,105], as well as control CREB activity [106]. DYRK3 has also been shown to be involved in controlling subcellular structure through LLPS (liquid-liquid phase separation) and stress granule (SG) and non-membrane bound organelle (NMO) generation in response to pro-aging cellular stress [107,108]. DYRK3 also possesses the capacity to link factors involved in nutrient sensing (mTORC1) factors to cellular protective effects linked to SG formation [63,109]. To further our investigation into how our evidence reveals the potential role of DYRK3 in the aging-disease nexus, we extracted the specific DYRK3-associated features from our original total aging-disease (1497 factor) protein cohort (Supplementary Figure S2). We found that, across every scale of inspection, the DYRK3 component found in the original aging-disease cohort was significantly greater than that expected randomly. The total number of aging-disease proteins common with our DYRK3 PPI signature was 73 proteins. This aging-disease-DYRK3 cohort was shown to be strongly associated with longevity regulation (Figure 9) and unexpectedly linked to regulatory modulation of the epidermal growth factor receptor (EGFR) (Figure 9 and Figure 10). This was an interesting finding as we have previously demonstrated that not only is the EGFR a subtle regulator of complex cell survival and proliferation activity [110,111,112] but also a key feature of classical neurometabolic aging [74,113,114]. In addition to this functional cooperation in the aging-disease nexus, it has also been demonstrated that DYRK3 may control EGFR functionality as well through a direct or indirect interaction process [67]. To reinforce and further investigate this connectivity, we characterized the intersection signatures of both human DYRK3 and EGFR (Figure 11). We found that the degree of functional intersection between DYRK3 and EGFR was considerably greater than that expected randomly, suggesting that this association is functionally relevant. Indeed, when assessing the functional sequelae of the 42, we found that the majority of the DYRK3-EGFR common PPI proteins generated a phenotype dominated by both EGFR functional regulation (‘EGFR tyrosine kinase inhibitor resistance’ [KEGG], ‘Downregulation of ERBB2 signaling’ [Reactome], ‘EGFR tyrosine kinase inhibitor resistance’ [WikiPathways]), and longevity/disease regulatory pathways (‘Longevity regulating pathway’ [KEGG], ‘Cellular Senescence’ [Reactome], ‘NAD metabolism, sirtuins and aging’ [WikiPathways]). This data suggests that the functional association of DYRK3 with EGFR signaling pathways has the potential to regulate the transition from pro-aging mechanisms to disease etiology. It is interesting to note that there is considerable (24 tissues: adipose; ovary; brain; colon; endometrium; heart; pancreas; prostate; kidney; testes; skin; stomach; thyroid; pancreatic beta cell; lung; urinary bladder; gall bladder; mammary gland; smooth muscle; coronary artery; trabecular bone tissue; adrenal gland; hippocampus; temporal lobe; hypothalamus) expression overlap between curated database profiles for both DYRK3 and EGFR (DYRK3 expression = profiles—https://www.bgee.org/gene/ENSG00000143479, accessed on 20 February 2025;—https://www.ebi.ac.uk/gxa/genes/ensg00000143479, accessed on 20 February 2025: EGFR expression profiles—https://www.bgee.org/gene/ENSG00000146648, accessed on 20 February 2025; https://www.ebi.ac.uk/gxa/genes/ensg00000146648, accessed on 20 February 2025). This considerable coordination of protein expression reinforces the potential for this interaction to occur in multiple tissues in the body. Hence, this association may possess activities across multiple physiological sites.

**Figure 12:**
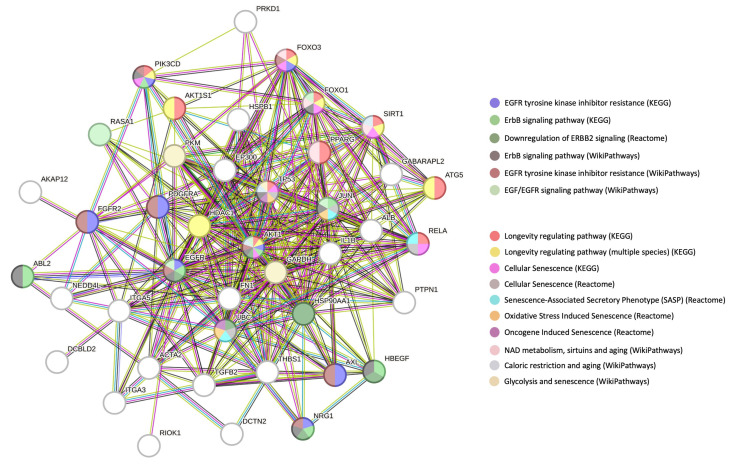
EGFR-DYRK3 functional nexus is associated with classical hallmarks of aging. STRING-based network creation with signaling pathway overlays was created for the specific 42 proteins identified as forming a functional nexus between EGFR and DYRK3. The network generated was created using the ‘evidence’ based setting allowing data to be collected from: textmining; curated experimental databases; gene neighborhood/fusions; gene co-occurrences; co-expression; protein homology. The color coding of the input nodes identifies the specific pathway enrichments (*p* < 0.05) found. The pathways employed for this include: KEGG; Reactome; WikiPathways. Protein nodes that are found in multiple pathway enrichments are denoted as multicolored according to the pathways they reside.

**Figure 13:**
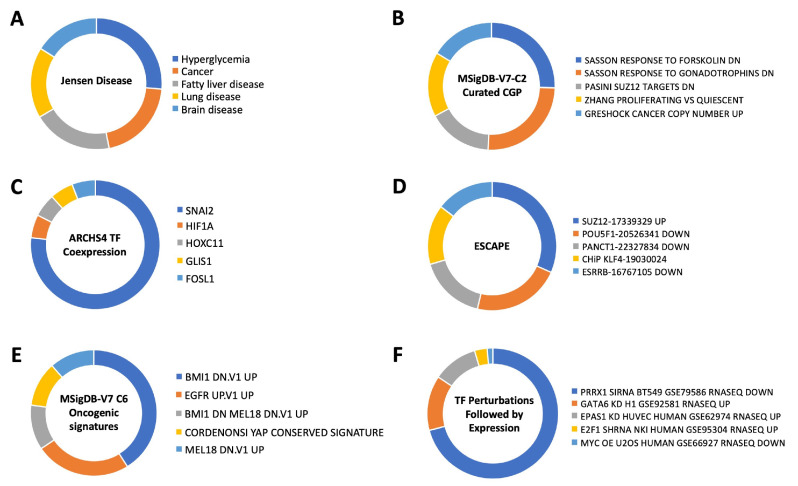
Multidimensional interrogation of the EGFR-DYRK3 functional nexus. Further enrichment analyses were performed on the 42 protein EGFR-DYRK3 aging nexus signature. Each pie chart depicts the top 5 most significantly enriched molecular pathway or dataset collection. (**A**) Jensen disease enrichment analysis indicates a broad spectrum of diseases that may be controlled by this aging-regulatory EGFR-DYRK3 nexus—indicating the fundamental basis of this molecular unit. (**B**) The CGP (Chemical Genetic Perturbation) dataset collection at MSigDB indicated an association of the EGFR-DYRK3 nexus with the Polycomb Repressive Complex 2 Subunit SUZ12. (**C**) ARCHS4 TF co-expression analysis demonstrated a potent association with SNAI2 that regulates cell fate in stressful circumstances. (**D**) ESCAPE database enrichment analysis reinforced the association of the EGFR-DYRK3 nexus with SUZ12. (**E**) MSigDB Oncogenic signature analysis revealed the importance of EGFR-DYRK3 with BMI1 and MEL18 that are also associated with SUZ12. (**F**) Analysis using the TF Perturbations Followed by Expression database revealed the importance of PRRX1 and the EGFR-DYRK3 nexus.

In addition to these aging-disease investigations, we further sought more nuanced aspects as to how this DYRK3-EGFR axis may exert its pathophysiological actions (Figure 12). Here, we found that this DYRK3-EGFR axis is associated with a broad range of diseases and may control cell fate (as well as stem cell fate) through control of the PRC2 complex via control of BMI1, MEL18 and SUZ12 (Figure 13). This complex has previously been shown to have a potent effect on the longevity of lower organisms such as *C. elegans* [115] as well as widespread disease prevention through the modulation of pro-aging cellular insults [116,117,118]. Given this data, it is reasonable to propose that chemical modulators of this novel DYRK3-EGFR axis may prove to be interesting novel agents with which to experimentally interdict aging-associated disease and damage.

We also assessed using human cell culture (HEK293) and ex vivo tissue analysis that EGFR and DYRK3 can associate with each other physically in a manner that is sensitive to both stressors that mimic aging (i.e., oxidative stress induced by hydrogen peroxide exposure: Supplementary Figure S3A,B) and also a natural advancing aging process (from ages of 3 to 12 months in murine cerebral cortex samples) (Supplementary Figure S3C). Hence, this physical demonstration reinforces the informatic definitions we have created thus far. In addition to these aging-disease investigations, we further sought more nuanced aspects as to how this DYRK3-EGFR axis may exert its pathophysiological actions (Figure 12). Here, we found that this DYRK3-EGFR axis is associated with a broad range of diseases and may control cell fate (as well as stem cell fate) through control of the PRC2 complex via control of BMI1, MEL18, and SUZ12 (Figure 13). This complex has previously been shown to significantly impact the longevity of lower organisms such as *C. elegans* [115], and to contribute to widespread disease prevention through the modulation of pro-aging cellular insults [116,117,118]. Given this data, it is reasonable to propose that chemical modulators of the DYRK3-EGFR axis may serve as promising agents to experimentally intervene in aging-associated disease and damage. While our data presents evidence of the potential impact of a DYRK3-EGFR interaction, it does not exclude the importance of many other protein-protein interactions in the aging-disease nexus. Our discovery process simply highlights a novel mechanism for investigating complex, multifactorial biomedical processes, such as the pathological aging process. Further research will potentially reveal linkages between this protein nexus and other factors linked to aging-disease pathomechanisms.

## Data Availability

All data pertaining to primary experimental result acquisition is made available by the included data Supplementary Figures S1 and S2, Supplementary Tables S1–S5.

## Supplementary Materials

The following supporting information can be downloaded at: https://www.mdpi.com/article/10.3390/cimb47030189/s1.
