# Supplementary material for: Unravelling Convergent Signaling Mechanisms Underlying the Aging-Disease Nexus Using Computational Language Analysis
Source: Curr Issues Mol Biol. 2025 Mar 14;47(3):189. doi: 10.3390/cimb47030189 (PMC11941692; doi:10.3390/cimb47030189)

Unravelling convergent signaling mechanisms underlying the aging-disease nexus using computational language analysis

Marina Junyent [1,2], Haki Noori [1,3], Robin De Schepper [1], Shanna Frajdenberg [1], Razan Khalid Abdullah Hussen Elsaigh [1], Patricia H. McDonald [4], Derek Duckett [5] and Stuart Maudsley [1,5*]

[1] Receptor Biology Lab, University of Antwerp, 2610 Wilrijk, Belgium
[2] IMIM, Hospital del Mar Research Institute, Barcelona, Spain
[3] KU Leuven, Oude Markt 13, 3000 Leuven, Belgium
[4] Lexicon Pharmaceuticals Inc. Research & Development, 2445 Technology Forest, The Woodlands, TX 77381, USA.
[5] H. Lee Moffitt Cancer Center, Department of Drug Discovery, 12902 Magnolia Drive, Tampa, FL 33612, USA.
* Correspondence: stuart.maudsley@moffitt.org. H. Lee Moffitt Cancer Center, Department of Drug Discovery, 12902 Magnolia Drive, Tampa, FL 33612, USA.

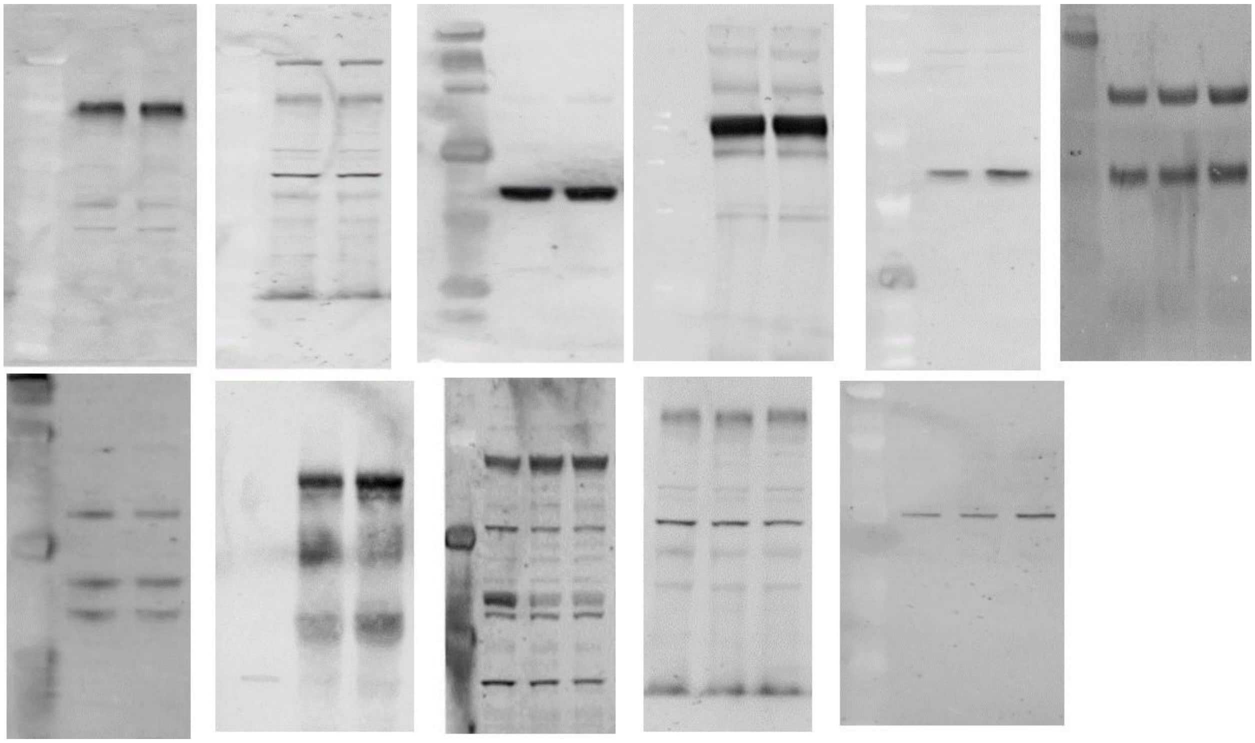

Supplement: Supplementary file 1 [file cimb-47-00189-s001.zip › Original-Images.pdf]
