# Supplementary material for: Unravelling Convergent Signaling Mechanisms Underlying the Aging-Disease Nexus Using Computational Language Analysis
Source: Curr Issues Mol Biol. 2025 Mar 14;47(3):189. doi: 10.3390/cimb47030189 (PMC11941692; doi:10.3390/cimb47030189)
Supplement: Supplementary file 1 [file cimb-47-00189-s001.zip › Supplemental-Figure-1.pdf]

Unravelling convergent signaling mechanisms underlying the aging-disease nexus using computational language analysis

Marina Junyent [1,2], Haki Noori [1,3], Robin De Schepper [1], Shanna Frajdenberg [1], Razan Khalid Abdullah Hussen Elsaigh [1], Patricia H. McDonald [4], Derek Duckett [5] and Stuart Maudsley [1,5*]

[1] Receptor Biology Lab, University of Antwerp, 2610 Wilrijk, Belgium
[2] IMIM, Hospital del Mar Research Institute, Barcelona, Spain
[3] KU Leuven, Oude Markt 13, 3000 Leuven, Belgium
[4] Lexicon Pharmaceuticals Inc. Research & Development, 2445 Technology Forest, The Woodlands, TX 77381, USA.
[5] H. Lee Moffitt Cancer Center, Department of Drug Discovery, 12902 Magnolia Drive, Tampa, FL 33612, USA.
* Correspondence: stuart.maudsley@moffitt.org. H. Lee Moffitt Cancer Center, Department of Drug Discovery, 12902 Magnolia Drive, Tampa, FL 33612, USA.

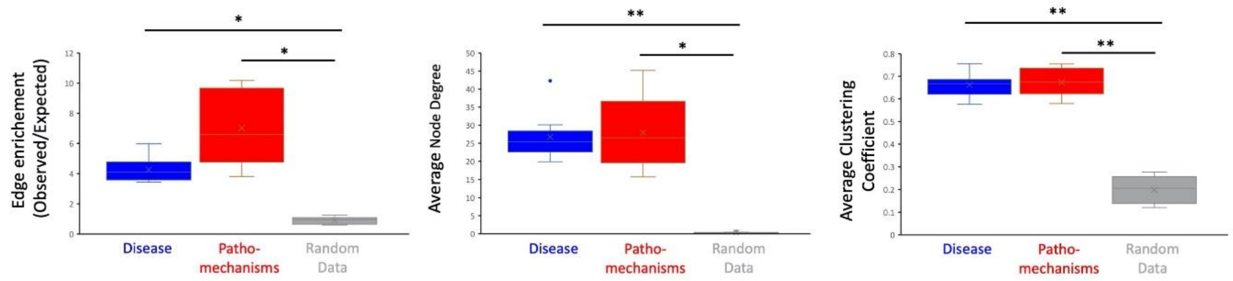

Supplementary Figure 1. Protein-protein interaction network data for disease and pathomechanism-related LLM data. (**A**) The STRING-derived edge enrichment scores are represented in Figure 1. Panel A indicates the box and whisker plots for disease, pathomechanism and random data analysis. Histogram bars represent the means ± SEM (standard error of the mean). The significance level is indicated in each figure as * *p*≤ 0.05; ** *p*≤ 0.01. (**B**) For the STRING-derived average node degree the following results were reported: CHD – 30.1; Cancer – 42.3; COPD – 27.8; Stroke – 25.3; AD – 25.5; T2DM – 19.9; CKD – 27.7; NAFLD – 23.8; Long-Covid – 22.7; MD – 22.4. For the STRING-derived average node degree the following results were reported: genomic instability – 42.2; telomere attrition – 45.2; disrupted epigenetic regulation – 15.7; disrupted proteostasis – 18; disrupted nutrient sensing – 20.2; mitochondrial dysfunction – 20.6; stem cell depletion – 30.1; disrupted cell-cell communication – 23.3; cell senescence – 29.7; cellular frailty – 34.7. For the STRING-derived average node degree the following results were reported: Random 1 – 0.324; Random 2 – 0.241; Random 3 – 0.313; Random 4 – 0.208; Random 5 – 0.312; Random 6 – 0.156; Random 7 – 0.152; Random 8 – 0.469; Random 9 – 0.278; Random 10 – 0.684. Histogram bars represent the means ± SEM (standard error of the mean). The significance level is indicated in each figure as * *p*≤ 0.05; ** *p*≤ 0.01. (**C**) The STRING-derived average clustering co-efficient for each of the diseases was reported as follows: CHD – 0.674; Cancer – 0.756; COPD – 0.707; Stroke – 0.668; AD – 0.623; T2DM – 0.615; CKD – 0.679; NAFLD – 0.663; Long-Covid – 0.667; MD – 0.576. The STRING-derived average clustering co-efficient for each of the pathomechanisms was reported as follows: genomic instability – 0.746; telomere attrition – 0.755; disrupted epigenetic regulation – 0.579; disrupted proteostasis – 0.635; disrupted nutrient sensing – 0.65; mitochondrial dysfunction – 0.588; stem cell depletion – 0.733; disrupted cell-cell communication – 0.635; cell senescence – 0.701; cellular frailty – 0.708. The STRING-derived average clustering co-efficient for each of the random networks was reported as follows: Random 1 – 0.216; Random 2 – 0.141; Random 3 – 0.277; Random 4 – 0.121; Random 5 – 0.195; Random 6 – 0.13; Random 7 – 0.152; Random 8 – 0.274; Random 9 – 0.228; Random 10 – 0.251. Histogram bars represent

the means ± SEM (standard error of the mean). The significance level is indicated in each figure as * $p \leq 0.05$; ** $p \leq 0.01$.
