# Supplementary material for: Unravelling Convergent Signaling Mechanisms Underlying the Aging-Disease Nexus Using Computational Language Analysis
Source: Curr Issues Mol Biol. 2025 Mar 14;47(3):189. doi: 10.3390/cimb47030189 (PMC11941692; doi:10.3390/cimb47030189)
Supplement: Supplementary file 1 [file cimb-47-00189-s001.zip › Supplemental-Figure-2.pdf]

Unravelling convergent signaling mechanisms underlying the aging-disease nexus using computational language analysis

Marina Junyent [1,2], Haki Noori [1,3], Robin De Schepper [1], Shanna Frajdenberg [1], Razan Khalid Abdullah Hussen Elsaigh [1], Patricia H. McDonald [4], Derek Duckett [5] and Stuart Maudsley [1,5]*

[1] Receptor Biology Lab, University of Antwerp, 2610 Wilrijk, Belgium
[2] IMIM, Hospital del Mar Research Institute, Barcelona, Spain
[3] KU Leuven, Oude Markt 13, 3000 Leuven, Belgium
[4] Lexicon Pharmaceuticals Inc. Research & Development, 2445 Technology Forest, The Woodlands, TX 77381, USA.
[5] H. Lee Moffitt Cancer Center, Department of Drug Discovery, 12902 Magnolia Drive, Tampa, FL 33612, USA.
* Correspondence: stuart.maudsley@moffitt.org. H. Lee Moffitt Cancer Center, Department of Drug Discovery, 12902 Magnolia Drive, Tampa, FL 33612, USA.

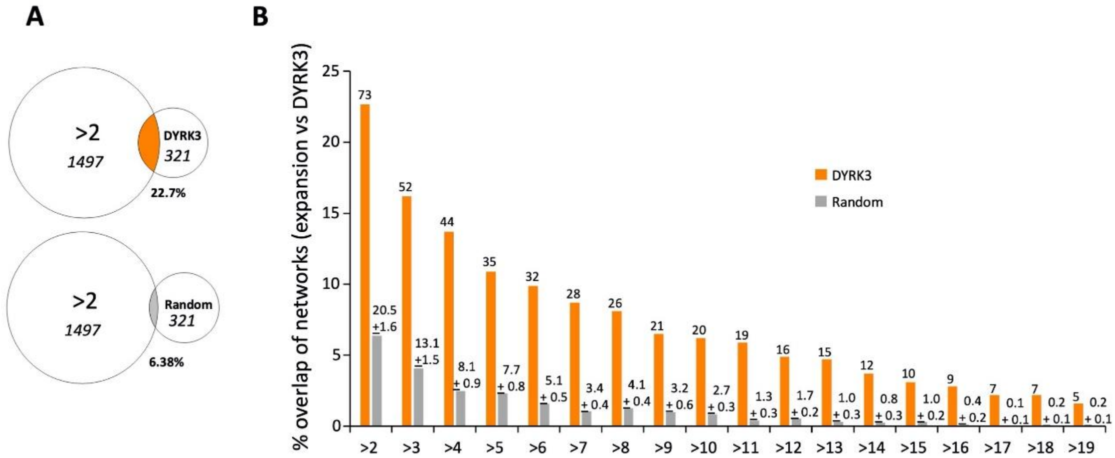

Supplementary Figure 2. Extraction of DYRK3-specific component of disease-pathomechanism core expansion. (**A**) A specific multi-database DYRK3-associated dataset (321 proteins) was investigated for its links to the disease-pathomechanism >2 dataset. 73 DYRK3-specific proteins were found in the disease-pathomechanism >2 dataset. When this was performed ten times with a similar magnitude random data set (321 proteins) a mean overlap of 20.5 +1.6 proteins was found. This 'random to real DYRK3' dataset investigation was performed with all analytical levels (>2 to >19) (**B**). At each analytical level there was a profound enrichment of DYRK3-associate proteins with disease-pathomechanism datasets, compared to random datasets of similar magnitudes (**B**).
