# Supplementary material for: Unravelling Convergent Signaling Mechanisms Underlying the Aging-Disease Nexus Using Computational Language Analysis
Source: Curr Issues Mol Biol. 2025 Mar 14;47(3):189. doi: 10.3390/cimb47030189 (PMC11941692; doi:10.3390/cimb47030189)
Supplement: Supplementary file 1 [file cimb-47-00189-s001.zip › Supplemental-Figure-3.pdf]

# Unravelling convergent signaling mechanisms underlying the aging-disease nexus using computational language analysis

**Marina Junyent [1,2], Haki Noori [1,3], Robin De Schepper [1], Shanna Frajdenberg [1], Razan Khalid Abdullah Hussen Elsaigh [1], Patricia H. McDonald [4], Derek Duckett [5], and Stuart Maudsley [1,5*]**

[1] Receptor Biology Lab, University of Antwerp, 2610 Wilrijk, Belgium
[2] IMIM, Hospital del Mar Research Institute, Barcelona, Spain
[3] KU Leuven, Oude Markt 13, 3000 Leuven, Belgium
[4] Lexicon Pharmaceuticals Inc. Research & Development, 2445 Technology Forest, The Woodlands, TX 77381, USA.
[5] H. Lee Moffitt Cancer Center, Department of Drug Discovery, 12902 Magnolia Drive, Tampa, FL 33612, USA.
* Correspondence: stuart.maudsley@moffitt.org. H. Lee Moffitt Cancer Center, Department of Drug Discovery, 12902 Magnolia Drive, Tampa, FL 33612, USA.

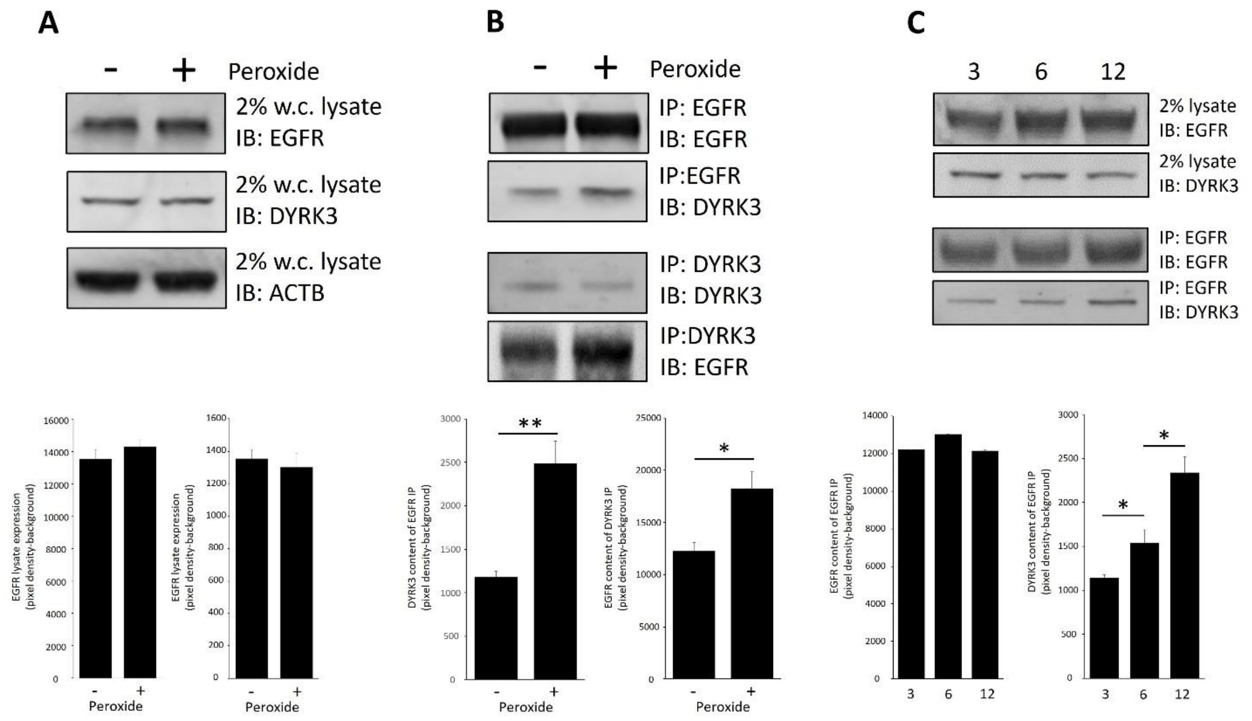


**Supplementary Figure 3.** Stress and age-dependent interactions between EGFR and DYRK3. (**A**) Total protein expression levels of EGFR or DYRK3 are not affected by an acute exposure to hydrogen peroxide. The associated histograms depict the mean $\pm$ standard error of the mean for n=3 individual experiments (this applies to all histograms in the Figure). (**B**) Acute hydrogen peroxide treatment of cells results in a significant increase of the DYRK3 content of EGFR-based immunecomplexes (p=0.0039) as well as the EGFR content of DYRK3 immunecomplexes (p=0.032). (**C**) EGFR and DYRK3 expression levels remain relatively constant in the murine cerebral cortex across three age timepoints (3, 6, 12 months). In contrast there is a significant (6 vs. 3 p=0.0284; 12 vs. 6 p=0.01306) age-associated increase in the DYRK3 content of EGFR immunecomplexes extracted from cortical tissues. IB = immunoblot, IP = Immunoprecipitation.
