# Supplementary material for: Unravelling Convergent Signaling Mechanisms Underlying the Aging-Disease Nexus Using Computational Language Analysis
Source: Curr Issues Mol Biol. 2025 Mar 14;47(3):189. doi: 10.3390/cimb47030189 (PMC11941692; doi:10.3390/cimb47030189)
Supplement: Supplementary file 1 [file cimb-47-00189-s001.zip › Supplemental-Table-1.pdf]

Table S1. LLM-generated datasets for major mortality causing human diseases. 100 protein identity datasets were generated for the following diseases: coronary heart disease (CHD); cancer; chronic obstructive pulmonary disease (COPD); stroke; Alzheimer's disease (AD); Type II diabetes mellitus (T2DM); chronic kidney disease (CKD); Non-alcoholic fatty liver disease (NAFLD); Long-Covid (also known as post-acute sequelae of SARS-CoV-2 infection (PASC)); major depression (MD).

**CHD**	**Cancer**	**COPD**	**Stroke**	**AD**	**T2DM**	**CKD**	**NAFLD**	**Long-COVID**	**MD**
ABCA1	ABL	ACE	ACE	ABCA7	ACC	ACE	ACADVL	ACE2	ABCB1
ACE	ABL1	ACE2	AGT	ADAM10	ADIPOQ	ACTH	ACC	ADM	ACE
ADIPOQ	AKT	AGER	AKT1	APOA1	Akt	ADIPOQ	ACOX1	ANXA2	AP1
ADM	AKT1	AKT1	ALOX15	APOB	AKT1	AGER	ACOX2	ANXA5	APAF1
AGT	AKT2	ALB	ALOX5	APOC1	AMPK	AKT1	ADFP	APP	BDNF
ALB	AKT3	ALOX5	APOE	APOC2	APOB	AMPK	ADIPOQ	ARL2BP	CACNA1C
ANXA5	ALK	BCL2	BCL2	APOC3	ATF6	AOC3	AHR	AT1R	CLOCK
APOA2	BAX	BCLXL	BDNF	APOE	CFTR	APOA1	AKT1	BDNF	CNR1
APOB	BCL2	BRAF	CASP3	APOJ	CPT1	APOB	AMPK	BIN2	COMT
APOE	BCL2L1	C2	CAT	APP	CRP	APOE	APOA1	BMP6	CREB1
AT1R	BCL6	CAT	COX1	BACE1	EIF2A	APRT	APOB	C1QA	CRH
AVP	BCR	CC16	COX2	BACE2	FABP4	AT1R	APOC3	C1QB	CRHBP
CALCA	BIRC5	CCL11	CRP	BDNF	FAS	AT2R	APOD	C1QC	CRHR1
CCL2	BRCA1	CCL2	EAAT2	BIN1	FASN	BMP7	APOF	C1RL	CRP
CCL3	BRCA2	CCL3	EDN1	CD2AP	FFAR1	CASR	APOJ	C3	CYP2C19
CCL4	CCNA1	CD4	EGFR	CD33	FOXO1	CAT	APOL1	CALR	CYP2C9
CCL4L1	CCNA2	CD8A	F10	CDK5	G6PC	CCL2	APOL2	CASP2	CYP2D6
CCL5	CCNB1	CDH1	F11	CLU	GCG	CDK20	APOL3	CCL11	DAOA
CETP	CCNB2	COX2	F12	CR1	GCK	CFH	APOM	CCL2	DAT
CKM	CCND1	CRP	F2	CRP	GH	COG2	APOML	CCL3	DBH
COG2	CCND2	CSF2	F5	CST3	GH1	COL1A1	BAX	CCL4	DISC1
CRLF1	CCND3	CTNNB1	F7	CTSB	GIPR	COL4A4	BCL2	CCL5	DRD1
CRP	CCNE1	CTSS	F8	CTSD	GLP1R	COX2	CASP3	CCR10	DRD2
CST3	CCNE2	CXCL10	F9	CTSH	GLUT4	CRH	CASP8	CCR19	DRD3
CXCL10	CCR5	CXCL8	FGF2	EPHA1	GRP	CRP	CAT	CCR2	DTNBP1
CXCL12	CD133	CYP1A1	FN	F10	GRP78	CST3	CD14	CCR21	EHD3
CYP2C19	CD44	CYP2B6	GABBR1	F11	GSK3	CTGF	CD36	CCR24	ESR1
DKK1	CDH1	CYP2D6	GABRR1	F12	GSK3B	EDN1	CPT1	CCR3	ESR2
EDN1	CDH2	CYP2E1	GDNF	F2	HERC2	EDNRA	CPT1A	CCR4	FGFR1
EPHX2	CDH3	CYP3A4	GLA	F5	HNF1A	EDNRB	CPT2	CCR5	FKBP4
F2	CDK2	ELANE	GPX1	F7	HNF1B	ENG	CRP	CCR7	FKBP5
FABP1	CDK4	FASL	GPX4	F8	HNF4A	EPO	CYCS	CCR8	FOSB
FABP3	CDK6	FGF2	GRIA4	FGF2	HSP27	FAN1	CYP2E1	CCR9	GABRA1
FABP4	CDKN2A	FN1	GRIN1	FYN	HSP60	FGF23	DGAT1	CD147	GABRA2
FGF23	CDKN2AIP	GCLC	GSK3B	GAPDH	HSP70	FLT1	DGAT2	CD40LG	GABRA3
GDF15	CDKN2C	GH1	GSTP1	GDNF	HSP90	FN1	FABP1	CD69	GABRA5

GHR	CDKN2D	GPX1	HBB	GFAP	IAPP	FOXO1	FABP2	CFB	GABRA6
GLUT4	CLDN1	GPX3	HMOX1	GPX1	IGF1	GCKR	FABP3	CFH	GABRB2
GRK2	CLDN3	GSR	HSP27	GSK3B	IL-1β	GH	FABP4	CFI	GABRG2
GRK5	CLDN4	GSTP1	HSP60	GST	IL6	GPX1	FABP5	CHMP1A	GAD1
HAVCR1	CLDN7	HDAC2	HSP70	HLA-DRB1	IL-6	HBB	FAS	CKMT1A	GAPDH
ICAM1	CXCR4	HMOX1	HSP90	HSP90AA1	INS	HIF1A	FASLG	CRP	GDNF
IGF1	E2F1	HSP70	HSP90AA1	HSPA1A	IRE1	HOGA1	FASN	CSF2	GNB3
IGFBP3	E2F2	HSP90	HSPA1A	HSPA5	IRS1	HSP27	FGF21	CXCL10	GRIA4
IL10	E2F3	IFNG	HSPA5	HSPA8	IRS2	HSP60	GCK	CXCL11	GRIN1
IL12	EGF	IL10	HSPB1	HSPB1	JAK2	HSP70	GLUT2	CXCL13	GRIN2A
IL17	EGFR	IL13	HSPD1	HSPD1	LEP	HSP90	GNMT	CXCL16	GRIN2B
IL18	EML4	IL17A	ICAM1	IDE	LXR	ICAM1	GSTM1	CXCL3	GRM5
IL1B	ERBB2	IL18	IL!7	IGF1	MAGEL2	IGF1	GSTP1	CXCL5	GRM7
IL23	ERBB3	IL1B	IL10	IL1B	MAPK1	IGF2	HADHA	CXCL8	GSK3B
IL33	ERRB4	IL1RN	IL13	IL6	MAPK11	IGFBP3	IL1B	CXCL9	HP
IL6	ESR1	IL2RA	IL18	INPP5D	MAPK3	IL18	IL6	EDN1	HSP90AA1
INS	ESR2	IL33	IL1B	ITGA3	MC4R	IL19	INS	EGF	HTR1A
IRS1	FGFR1	IL4	IL23	ITGB1	MKRN3	IL1B	INSR	EREG	HTR1B
ITIH4	FGFR2	IL5	IL27	ITGB3	MMP2	IL6	IRS1	FKBP1B	HTR2A
LCN2	FGFR3	IL6	IL33	LRP1	MMP9	INS	IRS2	FN1	HTR2C
LDHA	FLT3	IL8	IL37	MAPK1	NDN	INSR	JAK2	FRZB	IFNG
LDLR	FOS	KEAP1	IL4	MAPK3	NFKB1	IRS1	LDLR	FURIN	IL10
LEP	FOXO3	KLK15	IL6	MAPT	NF-κB	IRS2	LEP	GIPC3	IL1B
LGALS3	HER2	KRAS	JNK	MMP12	NPAP1	KL	LPGAT1	GIT1	IL6
LIPC	HIF1A	MAP2K1	MAPK1	MMP14	NPY	LCN2	LPL	GP5	KCNK2
LPL	HRAS	MM9	MAPK11	MMP2	PDI	LEP	MLYCD	GP6	KMO
MMP2	IGF1R	MMP12	MAPK2	MMP3	PDX1	LRP1	MTOR	HS6ST1	MAOA
MMP9	IGFBP3	MMP9	MCP1	MMP7	PIK3CA	MMP2	NAMPT	HSP27	MAOB
MPO	IGFBP5	MUC1	MMP9	MMP9	PIK3R1	MMP9	NFE2L2	HSP70	MAP2
NOS	JAK2	MUC13	NGF	NCAM1	PKCα	MT2A	NFKB1	HSP90	MBP
NOS3	JUN	MUC16	NOS1	NCSTN	PKCβ	MTOR	NR1H3	IFI10	MORC1
NOV	KRAS	MUC2	NOS2	NEP	PKCθ	NFKB1	NR1H4	IFNG	MTHFR
NPPA	KRT5	MUC5AC	NOS3	NGF	POMC	NGF	NR5A2	IL10	N3CR1
NPPB	KRT8	MUC5B	NPY	NOS2	PPARG	NOS1	PAK2	IL1B	NFKB1
P2RY12	MAPK1	NFE2L2	NR3C1	PDH	PTP1B	NOS2	PEMT	IL6	NPY
PCSK9	MAPK3	NLRP3	NR3C2	PICALM	PTPRC	NOX4	PI3K	LGALS3	NR3C1
PDGF	MAPK8	NOX4	NR4A1	PLAT	PTPRD	NOX5	PLIN2	LTA4H	NR3C2
PGE2	MCL1	NQO1	NR4A2	PON1	PTPRF	NOXA1	PNPLA3	MAP2K6	NTRK2
PGF	MDM2	NRAS	NR4A3	PRNP	PTPRJ	PDGF	PNPLA5	NLRP3	OXTR
PLA2G2A	MET	PDE4A	NTF3	PSEN1	PTPRN	PGE2	PPARA	NT5C3A	PCLO
PLAUR	MLL	PPARG	NTF4	PSEN2	PTPRO	PI3K	PPARD	PF4	PDLIM5
PPARA	MMP2	PRDX6	PDGF	PSENEN	PTPRS	PTH	PPARG	PLXNB3	POMC
PPARG	MMP9	PTPN11	PDGFR	PTGES	PTPRT	REN	PRKAA1	PPBP	PRKCA
PPP1R17	MYC	RAF1	PI3KCA	PTGES2	PTPRZ1	RETN	PRKAB1	RAB6A	PRKCD
PTAFR	NFKB1	REN	PLAT	PTGS2	RETN	SEC61A1	RBP4	RANTES	PRKCe

RBP4	NFKBIA	RIT1	PLAU	PTK2B	SERPINA7	SELE	RBP7	REN	PRKCz
REN	NRAS	SERPINA1	PRDX2	RTN4R	SIRT1	SELP	RETN	SELP	PTGS2
RETN	PDGF	SFTPA	PRKACA	SERPINA1	SLC2A1	SIRT1	RXRA	SEPTIN9	RELN
SCARB1	PDGFB	SFTPA2	PTGS1	SERPINA3	SLC2A4	SLC12A3	RXRG	SERPINE1	REN
SELE	PDGFRA	SFTPB	PTGS2	SERPINC1	SMAD4	SLC7A9	SIRT1	SKAP1	S100A10
SELL	PDGFRB	SFTPC	RHOA	SERPINE1	SNRPN	SNAI1	SLC27A1	SRC	S100B
SELP	PIK3CA	SFTPD	ROCK	SERPINF2	SREBP1	SOD1	SLC27A2	TACC3	SERT
SEPINE1	PTEN	SOD1	SERPINC1	SIRT1	SST	TGFB1	SLC27A3	TBC1D23	SLC6A15
SPP1	PTGS2	SOD2	SERPINE1	SOD1	STAT3	TIMP1	SOAT1	TLR3	SLC6A2
STUB1	RB1	SOD3	SERPINF2	SOD2	SULT2A1	TIMP2	SOAT2	TLR4	SLC6A4
TGFB	RET	SOS1	SLC2A10	SORL1	TANGO2	TLR2	SREBF1	TLR7	SOD1
TNF	SMAD4	TGFB	SOD1	TFAM	TCF7L2	TLR4	SREBP1	TLR8	SOD2
TNFA	TERT	TLR4	SOD2	TIMP1	TGFB1	TNFA	STAT3	TMEM106A	SST
TNNI3	TP53	TNF	SOD3	TIMP2	TIMP2	TWIST1	TGFB1	TMPRSS2	STK11
TNNT2	VEGF	TNFA	TAC1	TNF	TNFA	UTS2	TLR4	TNFA	TDO2
TXA2	VEGFA	TPS1	TNFA	TREM2	TNFα	VCAM1	TM6SF2	TREML1	TNF
VCAM1	VEGFR1	TRAC1	VCAM1	TUBB2A	TRIB3	VEGF	TNFA	VEGF	TP53
VEGF	VEGFR2	TRBC1	VEGFR	TUBB3	WFS1	VIM	TP53	VSIR	TPH1
WNT1	VIM	TXN2	VWF	VEGF	XBP1	VIP	XBP1	VWF	TPH2
