# Supplementary material for: Unravelling Convergent Signaling Mechanisms Underlying the Aging-Disease Nexus Using Computational Language Analysis
Source: Curr Issues Mol Biol. 2025 Mar 14;47(3):189. doi: 10.3390/cimb47030189 (PMC11941692; doi:10.3390/cimb47030189)
Supplement: Supplementary file 1 [file cimb-47-00189-s001.zip › Supplemental-Table-2.pdf]

Table S2. LLM-generated datasets for major aging-related pathomechanisms. 100 protein identity datasets were generated for the following aging pathomechanisms: genomic instability; telomere attrition; disrupted epigenetic regulation; disrupted proteostasis; disrupted nutrient sensing; mitochondrial dysfunction; stem cell depletion; disrupted cell-cell communication; cell senescence; cellular frailty.

Genomic Instability	Telomere Attrition	Disrupted Epigenetic Regulation	Disrupted Proteostasis	Disrupted Nutrient Sensing	Mitochondrial Dysfunction	Stem Cell Depletion	Disrupted Cell-Cell Communication	Cell Senescence	Cellular Frailty
53BP1	53BP1	ABHD8	AHSA1	ACC	AIFM1	OCT4	ADAM10	AMPK	AIM2
APEX1	ACD	AHRR	AMFR	ADIPOQ	APOB	AKT	ADAM17	ATM	AKT1
ATM	APEX1	AMER3	ANKRD2	ADIPOR1	ARID4B	ATM	ADAMTS4	ATR	AMPK
ATR	ATM	ASPA	ATF4	AKT1	BAD	ATR	ADAMTS5	BAX	ASF1
BLM	ATR	ATM	ATF6	AMPK	BAK	AXIN2	BDNF	BCL2	ATM
BLMIP	BLM	ATR	ATG101	ASNS	BAX	BMI1	BMP2	BLM	ATR
BRCA1	BRCA1	BLM	ATG12	ATGL	BCS1L	BRCA1	BMP4	BRCA1	BAX
BRCA2	BRCA2	BMI1	ATG13	BLOC1S1	CLPB	BRCA2	BMP6	BRCA2	BCL2
CENPF	CDC13	CACNG7	ATG18	CAMKK2	COX1	CCNA	BMP7	CASP3	BECN1
CHK2	CDC37	CBX7	ATG5	CAMKKB	COX10	CCNB1	CD44	CAT	BLM
DNA2	CDC45	CBX8	ATG7	CASR	COX4	CCND1	CD63	CCND1	BMI1
ERCC1	CDC6	CHK1	BAG3	CASTOR1	COX6C	CCND2	CD81	CCNE1	CAF-1
ERCC2	CDKN1A	CHK2	CALB1	CD36	CPT1B	CCND3	CD9	CDK4	CASP3
EXO1	CHK1	DNAJC2	CALR	CPT1A	CPT2	CCNE1	CDH1	CDK6	CASP8
FANCD2	CHK2	DNMT1	CANX	CPT1B	CRLS1	CCNG1	CDH17	CDKN1A	CASP9
FANCI	DDRGK1	DNMT3A	CASQ1	CPT2	CS	CCNH	CDH2	CDKN2A	CAT
FEN1	DKC1	DNMT3B	CCT1	CRTC1	CSF3	CD24	CDH3	CDKN2AIP	CD44
LIG1	DNA2	DPH7	CCT2	EIF2AK4	CYC1	CDK1	CDH5	CHK2	CDH12
LIG3	EXO1	E2F1	CCT3	FABP4	CYP27A1	CDK11	CFL2	CTGF	CDKN1A
MDC1	FANCD2	E2F3	CCT4	FABP5	CYPD	CDK2	CLDN1	DDB1	CDKN2A
MLH1	FANCI	E2F4	CCT5	FASN	DLD	CDK4	CLDN2	DDB2	CDKN2B
MRE11A	FEN1	E2F5	CCT6A	FATP1	DNM1L	CDK5	CLDN3	ERCC1	COX2
MSH2	H2AFX	E2F6	CCT6B	FATP4	ENDOG	CDK6	CLDN4	ERCC2	CTNNB1
MSH6	HMGB1	E2F7	CCT7	FFAR1	FASTKD2	CDK7	CLDN5	ERCC3	DDB2
NBS1	HMGB2	E2F8	CCT8	FFAR2	FH	CDK8	CTNNA	ERCC4	DNMT1
NEIL3	LIG1	EFCAB5	CLU	FFAR3	FIS1	CDK9	CTNNB	ERCC5	DNMT3A
PALB2	LIG3	EHMT2	CRCT1	FFAR4	FIS2	CDKN1A	CTNNG	ERK1	ERK2
PARP1	MCM2	ELOVL2	CRYAA	FGF21	FOXRED1	CDKN1B	EFNA1	ERK2	FAK
PARP2	MCM3	EZH2	CRYAB	FOXO1	GAMT	CDKN2A	EFNA2	FANCA	FLOT1
PARP3	MCM4	FANCD2	CTSB	GHRL	GATM	CDKN2B	EFNA3	FANCC	FOXO3
PARP4	MCM5	FHL2	CTSD	GLUT1	GLRX2	CDKN2C	EFNA4	FANCD2	GAS5
PIK3CA	MCM6	GATAD2A	CTSK	GLUT2	GPX1	CDKN2D	EFNB1	FANCG	GLUT1
PMS1	MCM7	GLIPR1L2	CTSL	GLUT3	HMOX1	CHK1	EFNB2	FEN1	GPX1
PMS2	MLH1	GOLGA6A	CTSS	GPR119	HSP60	CHK2	EFNB3	FGF21	GSK3B
POLB	MRE11	H2AC18	CTSV	GPR40	HSP70	DKK1	EFNB4	FOXO	HDAC1

POLD1	MRE11A	H2BC3	CTSZ	GPRC6A	HSP90	DKK2	EGF	GPX1	HIF1A
POLD2	MSH2	H3-5	EFEMP1	GSK3B	IDH1	DKK3	FGF10	HMOX1	HIRA
POLD3	MSH6	H4C1	EIF2AK3	HSL	MFF	DKK4	FGF2	HSP27	HOTAIR
POLD4	NBS1	HAT1	ERN1	IGF1	MFN1	DLL1	FGF7	HSP70	HP1
POLE	NHP2	HDAC1	FBLN5	IGF1R	MFN2	DLL3	FLNA	HSP90	HSP27
POLE2	NOP10	HDAC2	HSF1	INSR	MGME1	DLL4	FLNB	IGF1	HSP70
POLE3	ORC1	HDAC3	HSP70	IRS1	MPV17	E2F1	GDNF	IGFBP1	HSP90
POLE4	ORC2	HDAC4	HSP90	IRS2	NDUFA1	E2F2	GJA1	IGFBP2	IGF1
POLH	ORC3	HDAC6	HSPA5	LAMTOR1	NDUFA10	E2F3	HGF	IGFBP3	IGF1R
POLI	ORC4	HDAC8	HSPB8	LEP	NDUFA2	E2F4	ITGA1	IGFBP4	IL1B
POLK	PARP1	HIC1	LAMP2	LKB1	NDUFA8	E2F5	ITGA2	IGFBP5	IL6
PTCH1	PARP2	HMCES	MAPT	LPAR5	NDUFAF1	E2F6	ITGA3	IGFBP6	IRS4
PTEN	PARP3	HMGB1	MELTF	MIOS	NDUFAF3	E2F7	ITGA4	IGFBP7	JNK
RAD50	PARP4	HMGB2	MSRA	MLX	NDUFAF4	E2F8	ITGA5	IGFBP9	KEAP1
RAD51	PINX1	ITIH5	MYOZ1	MLXIPL	NDUFAF5	FOXO3	ITGA6	JNK	Ku70
RAD51B	PMS1	KDM2B	NPY4R	MTOR	NDUFS1	GNL3	ITGA7	KEAP1	Ku80
RAD51C	PMS2	KDM6B	NUP62	MTORC1	NDUFS2	H2AX	ITGB1	LIG1	LC3
RAD51D	POLA1	KL	PARK2	OGT	NDUFS3	HES1	ITGB2	LIG3	LMNA
RECQL4	POLB	KLF14	PDI	PI3K	NDUFS4	HES5	ITGB3	MAPK11	MALAT1
REV1	POLD1	KLHL35	PINK1	PIK3C3	NDUFS5	ID1	ITGB4	MLH1	MAPK11
REV3L	POLD2	KRTAP13-3	PNRC1	PLIN1	NDUFS6	ID2	ITGB5	MRE11	MAPKAPK3
RIF1	POLD3	LIG1	PPID	PLIN2	NDUFS7	ID3	ITGB6	MSH2	MMP14
RIF2	POLD4	LIG3	PPIL3	PLIN3	NDUFS8	ID4	ITGB7	MTOR	MTOR
RPA1	POLE	LMNA	PPP5C	PPARA	NDUFV1	JAGGED1	ITGB8	NBS1	NEAT1
RPA2	POLE2	LMNB1	PSMA1	PPARGC1A	NDUFV3	JAGGED2	MMP14	NKFB1	NFKB1
RPA3	POLE3	LSD1	PSMA2	PRAS40	NOS2	LGR5	MMP15	NRF1	NLRP3
RPA4	POLH	MAP1LC3C	PSMA3	PRKAA1	NUBPL	LIN28	MMP16	NRF2	NLRX1
RTEL1	POLI	MLH1	PSMA4	PRKAA2	OGDH	LIN28A	MMP17	PARP1	NOX4
SHFM1	POLK	MSH2	PSMA5	PRKAG1	OPA1	MDM2	MMP19	PARP2	NRF2
SLX4	POT1	MSH6	PSMA6	PRKAG2	PARKIN	MDM4	MMP2	PCNA	PARK7
SMARCAL1	POTIP1	NSUN4	PSMB1	PRKAR1A	PDHA1	METTL3	MMP24	PMS2	PARP1
SON	PRIM2	PCNA	PSMB10	PRKAR2B	PDP1	mTOR	MMP3	POLB	PDGF
SPRTN	PRKDC	PDE4C	PSMB2	PYY	PHB	MYC	MMP7	POLD1	PI3K
STAG2	RAD50	PFN3	PSMB3	RAB1B	PINK1	NANOG	MMP9	POLD2	PINK1
TERF2IP	RAD51	PMS2	PSMB4	RAP1A	POLG	NOTCH1	NGF	POLE	POT1
TIMELESS	RAD51B	PRAMEF2	PSMB5	RAP2C	PPARGC1A	NOTCH2	NRG1	POLG	PPARGC1A
TLK1	RAD51C	PRC1	PSMB6	RAPTOR	PRDX4	p73	NRG2	PPARGC1A	PRKN
TOP1	RAD51D	PRC2	PSMB7	RGS21	RRM2B	PIK3CA	NRG3	PRDX1	RAD51
TOP2A	RAD52	PRMT9	PSMB8	RHEB	SCO1	PIK3CD	NRG4	PRKDC	RAP1
TOP2B	RAD54	RB1	PSMB9	RORC	SCO2	PIK3CG	OCLN	RAD50	SIRT1
TP53	RAP1	RPA	PSMD5	RPTOR	SDHA	PIK3R1	PDGFA	RAD51	SIRT3
TP53BP1	RECQL4	SAMD10	PSME1	RRAGA	SDHAF1	PIK3R2	PDGFB	RAD52	SIRT4
TRIP13	RMI1	SIRT1	PSME2	RRAGB	SDHB	PIK3R3	PDGFC	RB	SIRT5
UBE2A	RMI2	SIRT2	PSME3	RRAGC	SDHC	POU5F1	PDGFD	RFC	SIRT6
UBE2B	RPA	SIRT3	PSME4	RRAGD	SDHD	PROM1	PFN2	RPA	SOD1

UBE2D1	RTEL1	SIRT4	PSME5	SIRT1	SELENOT	PTEN	SAMD9L	RTEL1	SOD2
UBE2D2	TCAB1	SIRT6	PSME6	SIRT3	SIRT3	RAD51	SDC1	SIRT1	SQSTM1
UBE2E1	TERC	SIRT7	RANBP2	SIRT6	SIRT5	RAD52	SDC2	SMAD	TERF2IP
UBE2N	TERF1	STK32A	RMDN3	SIRT7	SIRT6	RB1	SDC3	SOD1	TERT
UBE2T	TERF2	SUZ12	RNF126	SLC36A4	SLC17A5	SIRT1	SDC4	SOD2	TGFB1
UBR5	TERF2IP	TET1	RNF5	SLC38A9	SLC25A4	SIRT2	TGFB1	TERT	TIN2
USP1	TERRA	TET2	RNF6	SLC5A4	SLC25A6	SIRT3	TGFB2	TFIIH	TNFA
USP7	TERT	TET3	RORC	SREBP1C	SLC6A8	SIRT4	TGFB3	TGFA	TOMM20
VCP	TINF2	TRDMT1	SERPINI1	SZT2	SOD2	SIRT5	TIMP1	TGFB	TP53
WRN	TIPIN	TRIM58	SQSTM1	TAS1R1	SURF1	SIRT6	TIMP2	TP53	TRF1
XAB2	TP53	TRIM59	SRL	TAS1R2	TMEM70	SIRT7	TIMP3	TRF1	TRF2
XRCC1	TRF1	WRN	STUB1	TAS1R3	TOMM34	SOX2	TIMP4	TRF2	UCP2
XRCC2	TRF2	XRCC1	TRIM25	TAS2R38	TTC19	TCF7L1	TJP1	WRN	VDAC
XRCC3	WRN	XRCC5	TRIM27	TFEB	TXN1	TCF7L2	TJP2	XPA	VEGF
ZBTB44	XRCC2	XRCC6	UBC	TLR4	TXNRD2	TP53BP1	TJP3	XPC	WRAP53
ZBTB7A	XRCC3	ZFR2	UBD	TSC1	UCP1	TP63	TLN2	XPF	WRN
ZNF335	XRCC5	ZNF428	UCHL5	TSC2	UCP2	WNT1	VCL	XPG	XIST
ZNF687	XRCC6	ZNF488	ULK1	ULK1	UCP3	WNT3A	VEGFA	XRCC1	XRCC1
ZNF830	ZMYND11	ZNF518B	UPS	ULK2	UCP4	WNT5A	VEGFC	XRCC5	XRCC6
ZWINT	ZNF827	ZYG11A	VCP	WDR24	VDAC1	WNT7A	VEGFD	XRCC6	ZMPSTE24
