# Supplementary material for: Unravelling Convergent Signaling Mechanisms Underlying the Aging-Disease Nexus Using Computational Language Analysis
Source: Curr Issues Mol Biol. 2025 Mar 14;47(3):189. doi: 10.3390/cimb47030189 (PMC11941692; doi:10.3390/cimb47030189)
Supplement: Supplementary file 1 [file cimb-47-00189-s001.zip › Supplemental-Table-3.pdf]

Table S3. PubPular-generated datasets for major disease groups. 100 protein identity datasets were generated for the following diseases using PubPular semantic extraction: coronary heart disease (CHD); cancer; chronic obstructive pulmonary disease (COPD); stroke; Alzheimer's disease (AD); Type II diabetes mellitus (T2DM); chronic kidney disease (CKD); Non-alcoholic fatty liver disease (NAFLD); Long-Covid (also known as post-acute sequelae of SARS-CoV-2 infection (PASC)); major depression (MD).

CHD	Cancer	COPD	Stroke	AD	T2DM	CKD	NAFLD	Long-COVID	MD
ABCA1	ALK	ACE	ABCA2	ABCA7	ACE	ACE	ABHD5	AAK1	ACMSD
ABCG5	ARID1A	ACE2	ABCD2	ABI3	ACE2	ACP5	ACACA	ACE2	AP2B1
ABCG8	ASAH2B	ADAM33	ADAMTS13	ACHE	ADIPOQ	ACR	ACACB	ADAMTS13	AVPR1B
ACACA	ATXN7L1	ADRB2	AGAP5	ADAM10	AGER	ACTN4	ACOX1	AGT	BDNF
ACCS	BAP1	AHI1	AIF1	ADAMTS2	AGT	AGT	ACTA1	AGTR1	C3orf84
ACE	BARD1	ANGPT4	AP2B1	AGER	AGTR1	AGTR1	ADIPOQ	AMH	C4orf51
ACOD1	BEND2	ANGPTL3	AQP4	AGPS	AHSG	AHSG	AFP	ANGPT1	CCDC188
ADAMTS7	BRAF	AP2B1	BDNF	ANKRD66	ANGPT1	ALB	AHSG	ANGPT2	CCDC71
ADIPOQ	BRCA1	ARMC2	C10orf128	APBB1	ANGPT2	ANKS6	ALPP	ANGPT4	CINP
AGT	BRCA2	ARMC4	C11orf45	APH1A	ANGPT4	AP2B1	ANGPTL8	ANGPTL3	CNR1
ANGPTL3	BRIP1	ATP12A	C14orf79	APH1B	ANGPTL3	APOL1	APOB	ANKUB1	COMT
AP2B1	C2orf91	BMPR2	C17orf77	APOC1	ANGPTL8	APOM	APOC3	AP2B1	CRH
APOA1	C4orf19	CAPN15	CARF	APOE	AP2B1	AQP2	AWAT1	BSG	CRHBP
APOA5	C8orf82	CCDC114	CLDN5	APP	APLN	CASR	CCL4	BTNL2	CRHR1
APOB	C9orf84	CCDC151	CLPSL2	ATP11AUN	APOB	CCDC182	CD36	C11orf71	CSTA
APOC3	CATIP	CCDC38	COG2	B4DT89	CCL2	CD59	CIDEC	C19orf18	CYP2D6
APOC4	CCDC102A	CCDC39	COL4A1	BACE1	COG2	CD79A	CNBP	C9orf47	DCANP1
C10orf142	CCDC125	CCDC40	COL4A2	BACE2	CRP	CLEC14A	CPT1A	CCL3	DLG4
C1QTNF1	CCDC24	CCL11	CYP2C19	BCHE	CST3	COL4A3	CYP2E1	CMPK1	DRD2
C1QTNF9	CD274	CDHR3	DHRSX	BIN1	CYBB	COL4A4	CYP7A1	CRP	ERICH3
C8orf87	CD276	CENPJ	DKFZp313E1411	C10orf105	DBP	CRP	CYP8B1	CSTA	FAAH
CCDC126	CDK12	CFTR	DLGAP4	C9orf72	DPP4	CST3	DGAT1	CT47A1	FBXL15
CCDC92	CDK4	CHRNA3	DOCK3	CA1	DSPP	CUBN	DGAT2	CT47A1	FCHSD1
CELSR2	CDK6	CHRNA5	EHMT1	CA3	DUOX2	CYBRD1	DPP4	CTSL	FKBP5
CETP	CDX2	CHRNA6	EQTN	CA4	EDN1	CYP24A1	FABP1	CXCL10	FLJ00390
CHD1	CLDN18	CHRNB3	F10	CALHM1	EHMT1	CYP27B1	FABP4	CXorf57	FREM3
CHD2	CYP19A1	CHRNB4	F5	CASS4	ENHO	DBP	FASN	DDX58	GAD1
CHD9	EML4	COL4A5	F7	CD2AP	FGF21	DDAH1	FAT1	DPP9	GNPTAB
CHDH	ERBB2	CRP	FAM117B	CD33	FNDC5	DNTTIP2	FETUB	FAM106A	GRIA1
CMPK1	ERCC6L2	CYBB	FAM205A	CDK5	FOXO1	DPP4	FFAR2	FGB	GRIN2A
COG2	FAM135B	CYSLTR1	FAM47A	CDR1	G6PC2	EHMT1	FFAR3	FURIN	GRIN2B
CRP	FNIP2	DNAAF1	FBXL15	CDR2	GAD2	EPO	FGF19	FYCO1	GRM2
CST3	FOLH1	DNAAF2	FGB	CFAP99	GCG	EPOR	FGF21	GAD1	GRM3
DBP	FRG2C	DNAAF3	GLP1R	CHI3L1	GCGR	ESPN	FNDC5	GAGE7	GRM4
EDN1	GINM1	DNAAF5	GOLGA6A	CHMP2B	GCK	FABP1	FUT1	GAGE7	GRM5

ENHO	KAAG1	DNAH11	GP1BA	CLSTN1	GCKR	FGF23	GCG	GFY	HACD3
F7	KHDC1L	DNAH5	GPR37L1	CLU	GGT1	GC	GCKR	GGT1	HCRT
FABP3	KIAA0754	DNAI1	HTRA1	CR1	GIP	GFY	GGT1	GOLGA6L9	HIGD1C
FAM177B	KLRG2	DNAI2	ICA1L	CYP46A1	GIPR	GGT1	GGTLC1	GZMK	HOMER1
FGB	KRCC1	EDN1	KIAA1161	DOCK3	GLP1R	GLP1R	GIP	HIST1H3A	HTR1A
FGF23	KRT7	ELANE	KRBOX1	ECE2	GOLGA6A	GOLGA6A	GLP1R	IFIH1	HTR1B
GDF15	KRTAP10-12	FAM13A	LRCH1	EPHA1	GPT	HAMP	GPAM	IFITM2	HTR1D
GFY	KRTAP5-4	FOXJ1	MAS1	EXOC3L2	HBA1;	HAVCR1	GPBAR1	IFITM3	HTR2A
GGT1	LAG3	FXYD6-FXYD2	MCEMP1	FERMT2	IAPP	HLA-G	GPT	IFNA8	HTR2B
GLP1R	LIPT1	GNPTAB	MRS2	GLYATL3	IL12B	IGSF22	HSD17B13	IFNAR1	HTR2C
GOLGA6A	LRRC53	GSTCD	NAAA	GRN	IL18	KCNA10	INS	IFNAR2	HTR3A
HHIPL1	LRRD1	hCG_2045601	NBEAL1	HLA-DRB5	INS	KL	INSR	IFNE	HTR4
HMGCR	MB21D1	HYDIN	NCBP2L	IDE	INSM2	LCN2	IRS1	IFNK	HTR5A
ITPKC	MLH1	HYKK	NEFL	ITM2B	INSR	LRP2	KHK	IL12B	HTR6
KIAA1462	MSH2	IL13	NINJ2	LRP1	IRS1	MEPE	KLB	IL18	HTR7
KIF6	MSH6	IL17A	NOTCH3	LRP8	IRS2	MGEA5	KRT18	IL6R	IDO1
LDLR	MUC16	IL18	NOXRED1	MAMLD1	KCNJ11	MGP	LEP	IL7	INO80C
LGALS3	NAPSA	IL25	NPPB	MAPT	LEP	MSTN	LIPE	IRF7	KMO
LIPG	NECTIN4	IL33	NTF3	MCIDAS	LEPR	MYH9	LPIN1	LRRC74A	KYNU
LPL	NODAL	IL5	NUTM2A	MS4A4A	LPL	NAGLU	LPL	LTBP3	LHPP
MARCH2	NPEPPS	IL5RA	OCLN	MS4A6A	MAFA	NPHP1	LYPLAL1	LZTFL1	LRFN5
METRNL	NR4A1	IL6R	OR5H6	MS4A6E	MAS1L	NPHS1	MAP3K5	MAS1L	LTBP3
MIA3	NRAS	IREB2	P2RY12	MS4A8	MLXIPL	NPHS2	MARCH1	MASP2	MAOA
MRPS6	NTRK3	KCNMB2	PCDHB7	NCSTN	MME	NPIPB13	MBOAT7	MOG	MAOB
MYBPC3	OR10A3	KCNRG	PCSK9	NEFL	NAMPT	NPPB	MLXIPL	MRGPRE	NEGR1
MYH7	OR1S1	KRT74	PF4	NLRP5	NEUROG3	NR3C2	MTTP	MYOM2	NGF
NAAA	OR1S2	MMP12	PKNOX2	NME8	NFE2L2	PDILT	NAMPT	NAAA	NPY
NOS3	OR5W2	MPO	PLA2G7	NRGN	NKX6-1	PKD1	NCAN	NEFL	NR3C1
NPC1L1	OR8G2P	MSTN	PLAT	NYAP1	NLRP3	PKD2	NFE2L2	NLRP3	NTF3
NPPA	OTOS	MUC5AC	PMF1	OR5K3	NOS3	PKHD1	NLRP3	NRP1	NTF4
NPPB	PALB2	MUC5B	PMF1	OR5K4	NOX4	PLA2R1	NR1H3	OAS1	NTRK2
NR3C2	PDCD1	NLRP3	PPP1R21	PCDH11X	NPPB	POU1F1	NR1H4	OAS2	OPN1SW
OLR1	PGR	NPPB	PPP5D1	PHF1	NR3C2	PTH	PARVB	OAS3	OPRK1
OXCT1	PIK3CA	OR10A7	PROC	PICALM	P2RY12	RAPGEF5	PCK2	OR1D5	OPRM1
P2RY12	PLAG1	OR10P1	RASA4B	PILRA	PCSK9	RAPH1	PEMT	OR52N2	OR52I1
PCSK9	PLCXD2	OR2G3	RBPMS	PLD3	PDX1	REN	PLIN2	PF4	OXT
PHACTR1	PMS2	OR2T33	RNF213	PRNP	PNPLA3	RNLS	PLIN5	PHF1	OXTR
PLA2G7	PRAMEF10	ORMDL3	SCMH1	PSEN1	PPARA	SELENBP1	PNPLA2	PLBD1	PHF1
PLN	PRR18	PDE1C	SEC11A	PSEN2	PPARG	SHROOM3	PNPLA3	PPP1CA	PHF21B
PON1	PWWP2A	PDE4A	SELP	PSENEN	PPARGC1A	SLC20A2	PPARA	PROC	PMFBP1
PSRC1	RAD51C	PDE4C	SGCA	RIN3	PRKAA1	SLC22A11	PPARD	SH2D3A	POMC
RBM20	RAD51D	PDE5A	SH2D1A	RMDN1	PRKAA2	SLC22A12	PPARG	SLC22A31	PROX2
REN	RBM18	PDE7A	SH3PXD2A	SERPINA3	PRKAB1	SLC22A6	PPARGC1A	SLC6A19	SCLY
RETN	RNF43	PDZK1IP1	SLC25A44	SLC24A4	PTPRN	SLC22A8	PPP1R3B	SLC6A20	SLC1A2
SCAI	ROS1	PIH1D3	SLC5A2	SMUG1	RARRES2	SLC25A45	PRKAA1	SPECC1	SLC6A15

SELENBP1	SLTM	PRTN3	TCF24	SNCA	REN	SLC2A9	PRKAA2	SRPRA	SLC6A2
SELP	SMAGP	PSG5	TMEFF1	SORCS1	RETN	SLC34A1	PRKAB1	TCP11X2	SLC6A3
SERPINA5	SPATA2	PTGIR	TREX1	SORL1	SELENBP1	SLC34A3	PUM3	TLR3	SLC6A4
SLC5A2	SPATA6L	RNASE3	TSPAN10	SPDYE16	SERPINE1	SLC5A1	PYY	TLR7	SORCS3
SVEP1	TACSTD2	RSPH1	TSPAN19	SPDYE2B	SI	SLC5A2	RARRES2	TLR8	STAR
TAS2R50	TCP11X2	RSPH4A	TTC24	SPDYE3	SIRT1	SLC7A9	RETN	TM7SF2	TMEM161B
TET1	TIGIT	RSPH9	TTC7B	SPDYE5	SIRT3	SLC9A3	SAMM50	TMEM173	TMEM179
TMEM221	TMEM132C	SCGB1A1	VWA5B2	SV2A	SLC17A5	SLCO4C1	SAMSN1	TMEM81	TPH1
TMEM54	TMEM173	SERPINA1	VWDE	TARDBP	SLC2A2	SOST	SCD	TMPRSS2	TPH2
TMEM56-RWDD3	TMEM174	SFTPC	VWF	TMEM106B	SLC2A4	SPATA5L1	SIRT1	TMPRSS4	TPSG1
TNNI3	TMIGD3	SFTPD	ZBTB5	TNFSF10	SLC30A8	SYNPO	SLC17A5	TNFRSF8	TRANK1
TNNT1	TRABD2B	SGCA	ZCCHC14	TOMM40	SLC5A1	TF	SLC2A2	TRAPPC11	TTC9B
TNNT2	TRIM49C	SLC2A10	ZFHX3	TOMM40L	SLC5A2	THSD7A	SLC2A4	TUBA3C	UBE2Q2L
TSTD3	TRMT13	SPATA9	ZMYM2	TPSG1	SREBF1	TNFRSF11B	SLC5A2	UGT2A1	VGF
VCAM1	TTC34	THSD4	ZNF234	TREM2	TM6SF2	TRPV5	SREBF1	UGT2A1	VWDE
VPS51	TTF1	TMEM225	ZNF474	TREML2	TM7SF2	UCMA	SREBF2	VTN	WSCD1
VWF	TXK	TMPRSS2	ZNF551	UBE4A	TMPRSS2	UMOD	TLR4	VWF	ZKSCAN4
ZC3HC1	VGLL2	TPT1L_HUMAN	ZNF566	VSNL1	TXNIP	VDR	TM6SF2	ZFPM1	ZNF575
ZNF648	ZNF404	TSLP	ZNF700	WDR88	VCAM1	ZNF20	TMC4	ZGLP1	ZNF839
ZPR1	ZNF596	TST	ZNF790	ZCWPW1	WFS1	ZNF625	UCP1	ZNF524	ZSCAN9
