# Supplementary material for: Unravelling Convergent Signaling Mechanisms Underlying the Aging-Disease Nexus Using Computational Language Analysis
Source: Curr Issues Mol Biol. 2025 Mar 14;47(3):189. doi: 10.3390/cimb47030189 (PMC11941692; doi:10.3390/cimb47030189)
Supplement: Supplementary file 1 [file cimb-47-00189-s001.zip › Supplemental-Table-4.pdf]

Table S4. PubPular-generated datasets for major aging-related pathomechanisms. 100 protein identity datasets were generated for the following diseases using PubPular semantic extraction for the following aging pathomechanisms: genomic instability; telomere attrition; disrupted epigenetic regulation; disrupted proteostasis; disrupted nutrient sensing; mitochondrial dysfunction; stem cell depletion; disrupted cell-cell communication; cell senescence; cellular frailty.

**Genomic Instability**	**Telomere Attrition**	**Disrupted Epigenetic Regulation**	**Disrupted Proteostasis**	**Disrupted Nutrient Sensing**	**Mitochondrial Dysfunction**	**Stem Cell Depletion**	**Disrupted Cell-Cell Communication**	**Cell Senescence**	**Cellular Frailty**
ALK	ACD	ALKBH5	AHSA1	AKT1S1	ACSL4	ABCB5	ACTA1	ACAN	ACTN3
ARID1A	ACYP2	ARID1A	AMBRA1	AMBRA1	AIFM1	ABCG2	ACTA2	ACTA1	ACVR2B
ATM	ASF1A	ATM	AMFR	ATF4	ATF4	ACAN	ADAM10	ASPRV1	AGRN
ATR	ASPRV1	ATRX	ATF4	ATF6	ATF6	ACTN1	AGO2	ATG5	ARID4B
ATRIP	ATM	BAP1	ATF5	ATG10	ATG5	ALCAM	ANXA1	ATG7	ASPRV1
ATRX	ATR	BMI1	ATF6	ATG101	ATG7	ALDH1A1	ANXA2	ATM	B3GAT1
BAP1	ATRX	BRAF	ATG101	ATG12	ATP13A2	ANGPT1	ARF6	ATR	BPIFB4
BARD1	BICD1	BRCA1	ATG12	ATG13	BAK1	ASCL1	ARG1	BBC3	C12orf60
BLM	BLM	BRCA2	ATG13	ATG14	BAX	ATXN1	ARRDC1	BCL2L1	C16orf86
BRAF	BUB1B	BRD4	ATG14	ATG16L1	BECN1	BMI1	B4GALT3	BECN1	C1QTNF5
BRCA1	CBX3	CBX5	ATP13A2	ATG3	BNIP3	BMP1	CANX	BMI1	C6orf48
BRCA2	CBX5	CCND1	BAG1	ATG5	BNIP3L	BMP2	CAV1	CCNA2	C7orf73
BRIP1	CDKN2A	CD274	BAG3	ATG7	CALCOCO2	BMP4	CCL5	CCNB1	CD27
CCNE1	CHEK2	CDH1	BNIP3L	BECN1	CASP1	CD19	CCR2	CDK1	CD28
CD274	CLPTM1L	CDK4	C9orf72	BMT2	CASP9	CD24	CD163	CDK2	CD38
CDK12	CTC1	CDK6	CALCOCO2	BNIP3	CHCHD2	CD34	CD274	CDK4	CDKN2A
CDK4	DAXX	CDKN1A	CCPG1	C12orf66	COX4I1	CD38	CD3D	CDK6	CLOCK
CDK6	DCAF4	CDKN2A	CHCHD10	CALCOCO2	CPT1A	CD44	CD44	CDKN1A	CRP
CDKN2A	DKC1	CDKN2B	CLPP	CASTOR1	CYBB	CD63	CD47	CDKN2A	CRTC1
CHEK1	ERCC4	CREBBP	CLPX	CRTC1	CYCS	CD9	CD63	CDKN2B	CST3
CHEK2	ETV7	CTCF	CRYAB	CRTC2	DAPK2	CDH2	CD74	CHEK1	DNM1L
CTLA4	EXO1	CTNNB1	DNAJB1	DDIT4	DECR1	CR1	CD81	CHEK2	DOCK3
DNA2	FAM9A	DAPK1	DNAJB11	DEPTOR	DNM1L	CSF3	CD86	CXCL1	ELOVL2
DNMT1	FOXO4	DICER1	DNAJB6	EIF2A	DUOX2	CXCL12	CD9	CXCL12	FAM117A
DNMT3A	GAR1	DNMT1	DNAJC10	EIF2AK3	ERN1	CXCR4	CDH2	CXCR2	FAM46C
DNMT3B	GDF11	DNMT3A	DNM1L	EIF2AK4	FGF21	DCX	CDH5	DNMT1	FBXO32
ERCC4	H2AFJ	DNMT3B	EDEM1	EIF4EBP1	FIS1	EGF	CLDN5	E2F1	FFAR2
EXO1	H2AFX	DNMT3L	EIF2A	ERN1	FOXO3	EPCAM	COL1A1	EZH2	FFAR3
EZH2	HIRA	DOT1L	EIF2AK4	FFAR1	FUNDC1	FGF2	CSF1	FOXM1	FGF21
FANCA	KLRG1	E2F1	EIF2S1	FFAR2	GABPB1	FGF4	CTGF	FOXO1	FGF23
FANCD2	KRTAP13-3	EED	EIF2S2	FFAR3	GPX4	FGF7	CX3CL1	FOXO3	FIS1
FANCI	LIG3	EHMT2	ERN1	FFAR4	GSDMD	FLT3	CX3CR1	FOXO4	FNDC5
FANCM	LIG4	EP300	ERO1A	FLCN	HMOX1	FOXA2	CXCL1	GLB1	FOXO3
FBXW7	LMNB1	EZH1	FIS1	FNIP1	HSPA9	FUT1	CXCL12	GSK3A	FOXO4
H2AFX	LRRC34	EZH2	FUNDC1	FNIP2	KEAP1	GATA4	CXCL2	H2AFX	FST

IDH1	MAJIN	FOXA1	GABARAPL1	FOXO1	LRRK2	GDNF	CXCR2	HIF1A	GDF11
KMT2C	MB21D1	GDE1	GABARAPL2	FOXO3	MAP1LC3A	GFAP	EPCAM	HMGB1	GDF15
KMT2D	MDC1	HDAC1	HSF1	GABARAPL1	MAP1LC3B	HCST	FAP	HMOX1	GFRAL
KRAS	MRE11	HDAC2	HSP90B1	GABARAPL2	MCU	HES1	FLOT1	IFI27	GHR
LAG3	MUS81	HDAC3	HSPA5	GLP1R	MFF	HES3	GDE1	KEAP1	GHSR
LIG3	NAF1	HDAC4	HSPA8	ITFG2	MFN1	HGF	GJA1	KLF4	GRIP1
LIG4	NBN	HDAC5	HSPA9	LAMTOR1	MFN2	HLA-A	GJA4	LMNB1	hCG_2045601
LRP1B	NHP2	HDAC6	HSPB2	LAMTOR2	MIEF1	HOXB4	GJC1	MAP1LC3A	IGF1
MDC1	NOP10	HDAC9	HSPB8	LAMTOR4	MIEF2	INHBE	GPC1	MAP1LC3B	IL6R
MDM2	NRF1	IDH1	HTRA2	MAP1LC3A	MT-ATP6	ISL1	GZMB	MAPK14	KBTBD12
MLH1	OGG1	IDH2	HTT	MAP1LC3B	MT-ATP8	ITGA6	GZMK	MB21D1	KL
MLH3	PAPD5	IGF2	LONP1	MLST8	MT-ND1	ITGB1	HAVCR2	MDM2	KLRG1
MRE11	PARN	KDM1A	LRRK2	MTOR	MT-ND2	JAG1	HGF	MMP1	LMNB1
MSH2	PINX1	KDM5B	MCU	NBR1	MT-ND4	KITLG	HMGB1	MMP13	METTL21C
MSH3	POT1	KDM6A	MFF	NPRL2	MT-ND4L	KLF4	HNRNPA2B1	MMP3	MFN1
MSH6	PRAMEF2	KDM6B	MFN1	OGT	MT-ND5	LGR5	IDO1	MRE11	MFN2
MUS81	PRKDC	KLF4	MFN2	PDK1	MT-ND6	LIF	IL12B	MTOR	MSTN
MUTYH	RAD17	KMT2A	MIEF1	PIK3C3	NDUFB8	LIN28A	ITGA5	NANOG	MYF5
NBN	RAD50	KMT2B	MIEF2	PIK3R4	NFE2L2	LRIG1	ITGB1	NFE2L2	MYF6
NRAS	RAD52	KMT2C	NBR1	PINK1	NLRP3	MAP2	JAG1	NLRP3	MYH1
PALB2	RBBP8	KMT2D	NRF1	PPARGC1A	NOS3	MCAM	KAT2B	NOTCH1	MYLK4
PARP1	RECQL	KRAS	OMA1	PRKAA1	NOX1	MESP1	KLRK1	NOX4	MYOD1
PARP2	RECQL4	MBD2	OPA1	PRKAA2	NOX4	MRAP	LAG3	NT5E	MYOG
PDCD1	RECQL5	MDM2	OPTN	PRKAB1	NRF1	MSC	LGALS9	NUP62	NAMPT
PDCD1LG2	RIF1	MECP2	PARK7	PYY	NUP62	MSI1	MCAM	PARP1	NBPF6
PIK3CA	RPA1	METTL14	PARL	RAB7B	OMA1	MYH6	MIF	PIK3R1	NMRK1
PMS1	RTEL1	METTL3	PDIA3	RB1CC1	OPA1	MYH7	MRC1	PINK1	NRF1
PMS2	RUVBL1	MGMT	PINK1	RHEB	PARK7	MYL2	MZB1	POT1	NXT2
POLD1	RUVBL2	MLH1	PPP1R15A	RICTOR	PARL	MYL7	NKG7	POU5F1	OPA1
POLQ	SH3BP5	MSH2	PRKN	RORC	PDP1	NANOG	NLRP3	PPARGC1A	OR5B3
POT1	SIRT1	MSH6	PSMC4	RPS6KB1	PGAM5	NES	NOTCH1	PRKAA1	PAX7
PRKDC	SIRT2	MYC	PSMD11	RPTOR	PINK1	NEUROG2	NT5E	PRKAA2	PLIN5
PTEN	SIRT3	NANOG	PSMD12	RRAGA	POLG	NEUROG3	PANX1	PRKAB1	PPARGC1A
RAD50	SIRT4	NOTCH1	PSMD14	RRAGB	PPARA	NGFR	PDCD4	PRKDC	PRKAA1
RAD51	SIRT5	NRAS	PSME4	RRAGC	PPARGC1A	NKX2-5	PDCD6IP	RB1	PRKAA2
RAD51C	SIRT6	PIK3CA	RB1CC1	RRAGD	PPIF	NKX6-1	PECAM1	RELA	PRKAB1
RAD51D	SIRT7	POU5F1	RMDN3	SESN1	PRKAA1	NOG	PIK3R1	RUNX2	PRSS12
RAD52	SLX4	PRDM9	RPN1	SESN2	PRKAA2	NOTCH1	POSTN	SH3BP5	PUM3
RAD54L	SLX4IP	PTEN	SEL1L	SIRT1	PRKAB1	NRM	PTGDR	SIRT1	RENBP
RB1	SMARCAD1	RASSF1	SIRT3	SIRT3	PRKN	NT5E	RAB11A	SIRT2	RNASE11
RBBP4	SMARCAL1	RB1	SIRT4	SIRT4	RHOT1	OLIG2	RAB27A	SIRT3	RPS6KB1
RBBP8	STN1	RUNX1	SIRT5	SIRT6	RIPK3	OTX2	RAB27B	SIRT4	SAP25
RECQL	SUV39H1	RUNX3	SIRT6	SLC2A1	SIRT1	PAX6	RAB35	SIRT5	SARM1
RECQL4	SUV39H2	SETDB1	SIRT7	SLC2A2	SIRT3	PECAM1	RGS5	SIRT6	SCGB1D4
RIF1	TEN1	SIRT1	SQSTM1	SLC2A4	SIRT4	PLSCR4	SDCBP	SIRT7	SGCA

RNASEH1	TEP1	SIRT6	STIP1	SLC38A2	SIRT5	POU5F1	SMAD2	SMAD2	SIRT1
RNF43	TERF1	SMAD4	STUB1	SLC38A9	SIRT6	PROM1	SMAD3	SMAD3	SIRT2
RPA1	TERF2	SMARCA4	STX17	SLC7A5	SLC2A1	PTPRC	SMPD2	SOD2	SIRT3
RPA2	TERF2IP	SNAI1	SYVN1	SQSTM1	SLC2A4	REXO1	SMPD3	SOX2	SIRT4
SLX4	TERT	SOX2	TARDBP	STK11	SLC8B1	RSPO1	SOX9	SOX9	SIRT5
SMAD4	TINF2	SUV39H1	TFAM	STX17	SNCA	RUNX1	TGFA	STAT3	SIRT6
SMARCA4	TNKS	SUZ12	TFEB	TAS1R2	SOD1	RUNX2	THBS1	TERF1	SIRT7
SMARCAL1	TP53BP1	TDG	TIMM23	TAS1R3	SOD2	SHH	THY1	TERF2	SLC2A4
STK11	TPP1	TERT	TRAP1	TBC1D7	SQSTM1	SOX1	TIGIT	TERF2IP	SLC5A2
TERF2	TXN	TET1	UBQLN1	TFEB	TFAM	SOX17	TIMD4	TERT	SOST
TERT	UCP2	TET2	UBQLN2	TSC1	TFEB	SOX2	TJP1	TFEB	SULT2A1
TOPBP1	WRAP53	TET3	ULK1	TSC2	TIMM23	SOX9	TREM2	TGFA	TEX38
TP53	WRN	TP53	ULK2	ULK1	TOMM20	TGFB3	TSG101	THY1	TFAM
TP53BP1	XPA	TRDMT1	USP30	ULK2	TXN	THY1	TSPAN6	TINF2	TMEM8C
WEE1	XRCC5	TSC1	VAPB	UVRAG	TXNIP	TNFAIP6	TSPAN8	TMEM173	TNFAIP1
WRN	XRCC6	YAP1	VCP	WDR24	UCP2	TNNI1	VCAN	TP53	TNFRSF17
XRCC1	ZMPSTE24	YTHDC1	VDAC1	WDR59	ULK1	TNNT2	VPS4A	TP53BP1	TOM1L2
XRCC4	ZNF208	YTHDF1	VPS35	WIPI1	UQCRC2	WNT3A	VTA1	TXN	TRIM63
XRCC5	ZNF676	YTHDF2	XBP1	WIPI2	UTRN	WNT4	WNT5A	ULK1	TXN
XRCC6	ZSCAN4	ZEB1	YME1L1	XBP1	VDAC1	ZFP42	YAP1	YAP1	VDR
