# Supplementary material for: Unravelling Convergent Signaling Mechanisms Underlying the Aging-Disease Nexus Using Computational Language Analysis
Source: Curr Issues Mol Biol. 2025 Mar 14;47(3):189. doi: 10.3390/cimb47030189 (PMC11941692; doi:10.3390/cimb47030189)
Supplement: Supplementary file 1 [file cimb-47-00189-s001.zip › Supplemental-Table-5.pdf]

Table S5. Multilevel disease-pathomechanism nexus analysis. The differing levels of interactivity between major diseases and aging pathomechanisms are indicated by the different levels of connectivity from Level 1 to Level 5 (associated with Figure 5).

**Level 1**	**Level 2**	**Level 3**	**Level 4**	**Level 5**
ABCA1	ACC	ACC	ACC	AKT1
ABCA7	ACE	ADAM10	ADIPOQ	AMPK
ABCB1	ADAM10	ADIPOQ	AKT1	BAX
ABHD8	ADIPOQ	AKT	AMPK	BCL2
ABL	AKT	AKT1	APOB	CASP3
ABL1	AKT1	AMPK	BAX	CAT
ACADVL	AMPK	APOB	BCL2	GPX1
ACC	APOA1	ATF6	BDNF	GSK3B
ACD	APOB	BAX	BRCA1	HMOX1
ACE	APOE	BCL2	BRCA2	HSP27
ACE2	AT1R	BDNF	CASP3	HSP70
ACOX1	ATF6	BMP6	CAT	HSP90
ACOX2	ATM	BMP7	CCND1	IGF1
ACTH	ATR	BRCA1	CCNE1	MAPK11
ADAM10	BAX	BRCA2	CD44	MTOR
ADAM17	BCL2	CALR	CDH1	PIK3CA
ADAMTS4	BDNF	CASP3	CDK4	SIRT1
ADAMTS5	BLM	CASP8	CDK6	SOD1
ADFP	BMI1	CASR	CDKN2A	SOD2
ADIPOQ	BMP6	CAT	COX2	TGFB1
ADIPOR1	BMP7	CCNB1	CPT2	TP53
ADM	BRCA1	CCND1	E2F1	
AGER	BRCA2	CCND2	E2F3	
AGT	CALR	CCND3	EGF	
AHR	CASP3	CCNE1	FABP4	
AHRR	CASP8	CD36	FASN	
AHSA1	CASR	CD44	FGF2	
AIFM1	CAT	CDH1	FGF21	
AIM2	CCL2	CDH2	FOXO1	
AKT	CCL3	CDH3	FOXO3	
AKT1	CCNB1	CDK2	GDNF	
AKT2	CCND1	CDK4	GPX1	
AKT3	CCND2	CDK5	GSK3B	
ALB	CCND3	CDK6	HIF1A	
ALK	CCNE1	CDKN2A	HMOX1	
ALOX15	CD36	CDKN2AIP	HSP27	
ALOX5	CD44	CDKN2C	HSP60	
AMER3	CDH1	CDKN2D	HSP70	

AMFR	CDH2	CLDN1	HSP90	
AMPK	CDH3	CLDN3	HSPA5	
ANKRD2	CDK2	CLDN4	IGF1	
ANXA2	CDK4	CLU	IGF1R	
ANXA5	CDK5	COX1	IGFBP3	
AOC3	CDK6	COX2	IL1B	
AP1	CDKN1A	CPT1A	IL6	
APAF1	CDKN2A	CPT2	INSR	
APEX1	CDKN2AIP	CTGF	IRS1	
APOA1	CDKN2C	CTNNB1	IRS2	
APOA2	CDKN2D	CTSB	JNK	
APOB	CHK1	CTSD	KEAP1	
APOC1	CHK2	CTSS	LEP	
APOC2	CLDN1	DKK1	MAPK11	
APOC3	CLDN3	E2F1	MMP14	
APOD	CLDN4	E2F2	MMP2	
APOE	CLU	E2F3	MMP9	
APOF	COX1	EGF	MTOR	
APOJ	COX2	FABP4	NFKB1	
APOL1	CPT1A	FABP5	NGF	
APOL2	CPT2	FASN	NLRP3	
APOL3	CRP	FFAR1	NOS2	
APOM	CST3	FGF2	NOX4	
APOML	CTGF	FGF21	PDGF	
APP	CTNNB1	FOXO1	PIK3CA	
APRT	CTSB	FOXO3	PPARA	
ARID4B	CTSD	GDNF	PTEN	
ARL2BP	CTSS	GLUT2	RB1	
ASF1	CXCL10	GPX1	SIRT1	
ASNS	DKK1	GSK3B	SOD1	
ASPA	E2F1	HDAC2	SOD2	
AT1R	E2F2	HIF1A	TERT	
AT2R	E2F3	HMOX1	TGFB	
ATF4	EDN1	HSP27	TGFB1	
ATF6	EGF	HSP60	TIMP1	
ATG101	F2	HSP70	TIMP2	
ATG12	FABP4	HSP90	TLR4	
ATG13	FABP5	HSPA5	TNFA	
ATG18	FANCD2	IGF1	TP53	
ATG5	FASN	IGF1R	VEGF	
ATG7	FEN1	IGFBP3		
ATGL	FFAR1	IGFBP5		
ATM	FGF2	IL1B		
ATR	FGF21	IL6		
AVP	FN1	INSR		

AXIN2	FOXO1	IRS1		
BACE1	FOXO3	IRS2		
BACE2	GDNF	ITGA3		
BAD	GLUT2	ITGB1		
BAG3	GPX1	ITGB3		
BAK	GSK3B	JNK		
BAX	GSTP1	KEAP1		
BCL2	HDAC2	KL		
BCL2L1	HIF1A	LEP		
BCL6	HMOX1	MAPK11		
BCLXL	HSP27	MAPT		
BCR	HSP60	MDM2		
BCS1L	HSP70	MMP14		
BDNF	HSP90	MMP2		
BECN1	HSP90AA1	MMP3		
BIN1	HSPA5	MMP7		
BIN2	ICAM1	MMP9		
BIRC5	IFNG	MTOR		
BLM	IGF1	MYC		
BLMIP	IGF1R	NFKB1		
BLOC1S1	IGFBP3	NGF		
BMI1	IGFBP5	NLRP3		
BMP2	IL10	NOS2		
BMP4	IL18	NOX4		
BMP6	IL1B	PDGF		
BMP7	IL33	PDGFB		
BRAF	IL6	PDI		
BRCA1	INS	PIK3CA		
BRCA2	INSR	PIK3R1		
C1QA	IRS1	PLIN2		
C1QB	IRS2	PPARA		
C1QC	ITGA3	PRKAA1		
C1RL	ITGB1	PTEN		
C2	ITGB3	RB1		
C3	JAK2	SIRT1		
CACNA1C	JNK	SOD1		
CACNG7	KEAP1	SOD2		
CAF-1	KL	STUB1		
CALB1	LEP	TCF7L2		
CALCA	LIG1	TERT		
CALR	LIG3	TGFB		
CAMKK2	MAPK1	TGFB1		
CAMKKB	MAPK11	TIMP1		
CANX	MAPK3	TIMP2		
CASP2	MAPT	TLR4		

CASP3	MDM2	TNFA		
CASP8	MLH1	TP53		
CASP9	MMP14	VEGF		
CASQ1	MMP2	VEGFA		
CASR	MMP3	WNT1		
CASTOR1	MMP7			
CAT	MMP9			
CBX7	MSH2			
CBX8	MSH6			
CC16	MTOR			
CCL11	MYC			
CCL2	NBS1			
CCL3	NFKB1			
CCL4	NGF			
CCL4L1	NLRP3			
CCL5	NOS2			
CCNA	NOX4			
CCNA1	NPY			
CCNA2	PARP1			
CCNB1	PARP2			
CCNB2	PDGF			
CCND1	PDGFB			
CCND2	PDI			
CCND3	PIK3CA			
CCNE1	PIK3R1			
CCNE2	PINK1			
CCNG1	PLIN2			
CCNH	PMS2			
CCR10	POLB			
CCR19	POLD1			
CCR2	POLD2			
CCR21	POLE			
CCR24	PPARA			
CCR3	PPARG			
CCR4	PPARGC1A			
CCR5	PRKAA1			
CCR7	PTEN			
CCR8	PTGS2			
CCR9	RAD50			
CCT1	RAD51			
CCT2	RAD52			
CCT3	RB1			
CCT4	REN			
CCT5	RETN			
CCT6A	RPA			

CCT6B	RTEL1			
CCT7	SELP			
CCT8	SERPINE1			
CD133	SIRT1			
CD14	SIRT3			
CD147	SIRT4			
CD24	SIRT5			
CD2AP	SIRT6			
CD33	SIRT7			
CD36	SOD1			
CD4	SOD2			
CD40LG	STUB1			
CD44	TCF7L2			
CD63	TERF2IP			
CD69	TERT			
CD81	TGFB			
CD8A	TGFB1			
CD9	TIMP1			
CDC13	TIMP2			
CDC37	TLR4			
CDC45	TNF			
CDC6	TNFA			
CDH1	TP53			
CDH12	TRF1			
CDH17	TRF2			
CDH2	VCAM1			
CDH3	VEGF			
CDH5	VEGFA			
CDK1	WNT1			
CDK11	WRN			
CDK2	XRCC1			
CDK20	XRCC5			
CDK4	XRCC6			
CDK5				
CDK6				
CDK7				
CDK8				
CDK9				
CDKN1A				
CDKN1B				
CDKN2A				
CDKN2AIP				
CDKN2B				
CDKN2C				
CDKN2D				

CENPF				
CETP				
CFB				
CFH				
CFI				
CFL2				
CFTR				
CHK1				
CHK2				
CHMP1A				
CKM				
CKMT1A				
CLDN1				
CLDN2				
CLDN3				
CLDN4				
CLDN5				
CLDN7				
CLOCK				
CLPB				
CLU				
CNR1				
COG2				
COL1A1				
COL4A4				
COMT				
COX1				
COX10				
COX2				
COX4				
COX6C				
CPT1				
CPT1A				
CPT1B				
CPT2				
CR1				
CRCT1				
CREB1				
CRH				
CRHBP				
CRHR1				
CRLF1				
CRLS1				
CRP				
CRTC1				

CRYAA				
CRYAB				
CS				
CSF2				
CSF3				
CST3				
CTGF				
CTNNA				
CTNNB				
CTNNB1				
CTNNG				
CTSB				
CTSD				
CTSH				
CTSK				
CTSL				
CTSS				
CTSV				
CTSZ				
CXCL10				
CXCL11				
CXCL12				
CXCL13				
CXCL16				
CXCL3				
CXCL5				
CXCL8				
CXCL9				
CXCR4				
CYC1				
CYCS				
CYP1A1				
CYP27A1				
CYP2B6				
CYP2C19				
CYP2C9				
CYP2D6				
CYP2E1				
CYP3A4				
CYPD				
DAOA				
DAT				
DBH				
DDB1				
DDB2				

DDRGK1				
DGAT1				
DGAT2				
DISC1				
DKC1				
DKK1				
DKK2				
DKK3				
DKK4				
DLD				
DLL1				
DLL3				
DLL4				
DNA2				
DNAJC2				
DNM1L				
DNMT1				
DNMT3A				
DNMT3B				
DPH7				
DRD1				
DRD2				
DRD3				
DTNBP1				
E2F1				
E2F2				
E2F3				
E2F4				
E2F5				
E2F6				
E2F7				
E2F8				
EAAT2				
EDN1				
EDNRA				
EDNRB				
EFCAB5				
EFEMP1				
EFNA1				
EFNA2				
EFNA3				
EFNA4				
EFNB1				
EFNB2				
EFNB3				

EFNB4				
EGF				
EGFR				
EHD3				
EHMT2				
EIF2A				
EIF2AK3				
EIF2AK4				
ELANE				
ELOVL2				
EML4				
ENDOG				
ENG				
EPHA1				
EPHX2				
EPO				
ERBB2				
ERBB3				
ERCC1				
ERCC2				
ERCC3				
ERCC4				
ERCC5				
EREG				
ERK1				
ERK2				
ERN1				
ERRB4				
ESR1				
ESR2				
EXO1				
EZH2				
F10				
F11				
F12				
F2				
F5				
F7				
F8				
F9				
FABP1				
FABP2				
FABP3				
FABP4				
FABP5				

FAK				
FAN1				
FANCA				
FANCC				
FANCD2				
FANCG				
FANCI				
FAS				
FASL				
FASLG				
FASN				
FASTKD2				
FATP1				
FATP4				
FBLN5				
FEN1				
FFAR1				
FFAR2				
FFAR3				
FFAR4				
FGF10				
FGF2				
FGF21				
FGF23				
FGF7				
FGFR1				
FGFR2				
FGFR3				
FH				
FHL2				
FIS1				
FIS2				
FKBP1B				
FKBP4				
FKBP5				
FLNA				
FLNB				
FLOT1				
FLT1				
FLT3				
FN				
FN1				
FOS				
FOSB				
FOXO				

FOXO1				
FOXO3				
FOXRED1				
FRZB				
FURIN				
FYN				
G6PC				
GABBR1				
GABRA1				
GABRA2				
GABRA3				
GABRA5				
GABRA6				
GABRB2				
GABRG2				
GABRR1				
GAD1				
GAMT				
GAPDH				
GAS5				
GATAD2A				
GATM				
GCG				
GCK				
GCKR				
GCLC				
GDF15				
GDNF				
GFAP				
GH				
GH1				
GHR				
GHRL				
GIPC3				
GIPR				
GIT1				
GJA1				
GLA				
GLIPR1L2				
GLP1R				
GLRX2				
GLUT1				
GLUT2				
GLUT3				
GLUT4				

GNB3				
GNL3				
GNMT				
GOLGA6A				
GP5				
GP6				
GPR119				
GPR40				
GPRC6A				
GPX1				
GPX3				
GPX4				
GRIA4				
GRIN1				
GRIN2A				
GRIN2B				
GRK2				
GRK5				
GRM5				
GRM7				
GRP				
GRP78				
GSK3				
GSK3B				
GSR				
GST				
GSTM1				
GSTP1				
H2AC18				
H2AFX				
H2AX				
H2BC3				
H3-5				
H4C1				
HADHA				
HAT1				
HAVCR1				
HBB				
HDAC1				
HDAC2				
HDAC3				
HDAC4				
HDAC6				
HDAC8				
HER2				

HERC2				
HES1				
HES5				
HGF				
HIC1				
HIF1A				
HIRA				
HLA-DRB1				
HMCES				
HMGB1				
HMGB2				
HMOX1				
HNF1A				
HNF1B				
HNF4A				
HOGA1				
HOTAIR				
HP				
HP1				
HRAS				
HS6ST1				
HSF1				
HSL				
HSP27				
HSP60				
HSP70				
HSP90				
HSP90AA1				
HSPA1A				
HSPA5				
HSPA8				
HSPB1				
HSPB8				
HSPD1				
HTR1A				
HTR1B				
HTR2A				
HTR2C				
IAPP				
ICAM1				
ID1				
ID2				
ID3				
ID4				
IDE				

IDH1				
IFI10				
IFNG				
IGF1				
IGF1R				
IGF2				
IGFBP1				
IGFBP2				
IGFBP3				
IGFBP4				
IGFBP5				
IGFBP6				
IGFBP7				
IGFBP9				
IL10				
IL12				
IL13				
IL17				
IL17A				
IL18				
IL19				
IL1B				
IL-1B				
IL1RN				
IL23				
IL27				
IL2RA				
IL33				
IL37				
IL4				
IL5				
IL6				
IL-6				
IL8				
INPP5D				
INS				
INSR				
IRE1				
IRS1				
IRS2				
IRS4				
ITGA1				
ITGA2				
ITGA3				
ITGA4				

ITGA5				
ITGA6				
ITGA7				
ITGB1				
ITGB2				
ITGB3				
ITGB4				
ITGB5				
ITGB6				
ITGB7				
ITGB8				
ITIH4				
ITIH5				
JAGGED1				
JAGGED2				
JAK2				
JNK				
JUN				
KCNK2				
KDM2B				
KDM6B				
KEAP1				
KL				
KLF14				
KLHL35				
KLK15				
KMO				
KRAS				
KRT5				
KRT8				
KRTAP13-3				
Ku70				
Ku80				
LAMP2				
LAMTOR1				
LC3				
LCN2				
LDHA				
LDLR				
LEP				
LGALS3				
LGR5				
LIG1				
LIG3				
LIN28				

LIN28A				
LIPC				
LKB1				
LMNA				
LMNB1				
LPAR5				
LPGAT1				
LPL				
LRP1				
LSD1				
LTA4H				
LXR				
MAGEL2				
MALAT1				
MAOA				
MAOB				
MAP1LC3C				
MAP2				
MAP2K1				
MAP2K6				
MAPK1				
MAPK11				
MAPK2				
MAPK3				
MAPK8				
MAPKAPK3				
MAPT				
MBP				
MC4R				
MCL1				
MCM2				
MCM3				
MCM4				
MCM5				
MCM6				
MCM7				
MCP1				
MDC1				
MDM2				
MDM4				
MELTF				
MET				
METTL3				
MFF				
MFN1				

MFN2				
MGME1				
MIOS				
MKRN3				
MLH1				
MLL				
MLX				
MLXIPL				
MLYCD				
MM9				
MMP12				
MMP14				
MMP15				
MMP16				
MMP17				
MMP19				
MMP2				
MMP24				
MMP3				
MMP7				
MMP9				
MORC1				
MPO				
MPV17				
MRE11				
MRE11A				
MSH2				
MSH6				
MSRA				
MT2A				
MTHFR				
MTOR				
MTORC1				
MUC1				
MUC13				
MUC16				
MUC2				
MUC5AC				
MUC5B				
MYC				
MYOZ1				
N3CR1				
NAMPT				
NANOG				
NBS1				

NCAM1				
NCSTN				
NDN				
NDUFA1				
NDUFA10				
NDUFA2				
NDUFA8				
NDUFAF1				
NDUFAF3				
NDUFAF4				
NDUFAF5				
NDUFS1				
NDUFS2				
NDUFS3				
NDUFS4				
NDUFS5				
NDUFS6				
NDUFS7				
NDUFS8				
NDUFV1				
NDUFV3				
NEAT1				
NEIL3				
NEP				
NFE2L2				
NFKB1				
NFKBIA				
NGF				
NHP2				
NKFB1				
NLRP3				
NLRX1				
NOP10				
NOS				
NOS1				
NOS2				
NOS3				
NOTCH1				
NOTCH2				
NOV				
NOX4				
NOX5				
NOXA1				
NPAP1				
NPPA				

NPPB				
NPY				
NPY4R				
NQO1				
NR1H3				
NR1H4				
NR3C1				
NR3C2				
NR4A1				
NR4A2				
NR4A3				
NR5A2				
NRAS				
NRF1				
NRF2				
NRG1				
NRG2				
NRG3				
NRG4				
NSUN4				
NT5C3A				
NTF3				
NTF4				
NTRK2				
NUBPL				
NUP62				
OCLN				
OGDH				
OGT				
OPA1				
ORC1				
ORC2				
ORC3				
ORC4				
OXTR				
P2RY12				
p73				
PAK2				
PALB2				
PARK2				
PARK7				
PARKIN				
PARP1				
PARP2				
PARP3				

PARP4				
PCLO				
PCNA				
PCSK9				
PDE4A				
PDE4C				
PDGF				
PDGFA				
PDGFB				
PDGFC				
PDGFD				
PDGFR				
PDGFRA				
PDGFRB				
PDH				
PDHA1				
PDI				
PDLIM5				
PDP1				
PDX1				
PEMT				
PF4				
PFN2				
PFN3				
PGE2				
PGF				
PHB				
PI3KCA				
PICALM				
PIK3C3				
PIK3CA				
PIK3CD				
PIK3CG				
PIK3R1				
PIK3R2				
PIK3R3				
PINK1				
PINX1				
PKCB				
PLA2G2A				
PLAT				
PLAU				
PLAUR				
PLIN1				
PLIN2				

PLIN3				
PLXNB3				
PMS1				
PMS2				
PNPLA3				
PNPLA5				
PNRC1				
POLA1				
POLB				
POLD1				
POLD2				
POLD3				
POLD4				
POLE				
POLE2				
POLE3				
POLE4				
POLG				
POLH				
POLI				
POLK				
POMC				
PON1				
POT1				
POTIP1				
POU5F1				
PPARA				
PPARD				
PPARG				
PPARGC1A				
PPBP				
PPID				
PPIL3				
PPP1R17				
PPP5C				
PRAMEF2				
PRAS40				
PRC1				
PRC2				
PRDX1				
PRDX2				
PRDX4				
PRDX6				
PRIM2				
PRKAA1				

PRKAA2				
PRKAB1				
PRKACA				
PRKAG1				
PRKAG2				
PRKAR1A				
PRKAR2B				
PRKCA				
PRKCD				
PRKCE				
PRKCZ				
PRKDC				
PRKN				
PRMT9				
PRNP				
PROM1				
PSEN1				
PSEN2				
PSENEN				
PSMA1				
PSMA2				
PSMA3				
PSMA4				
PSMA5				
PSMA6				
PSMB1				
PSMB10				
PSMB2				
PSMB3				
PSMB4				
PSMB5				
PSMB6				
PSMB7				
PSMB8				
PSMB9				
PSMD5				
PSME1				
PSME2				
PSME3				
PSME4				
PSME5				
PSME6				
PTAFR				
PTCH1				
PTEN				

PTGES				
PTGES2				
PTGS1				
PTGS2				
PTH				
PTK2B				
PTP1B				
PTPN11				
PTPRC				
PTPRD				
PTPRF				
PTPRJ				
PTPRN				
PTPRO				
PTPRS				
PTPRT				
PTPRZ1				
PYY				
RAB1B				
RAB6A				
RAD50				
RAD51				
RAD51B				
RAD51C				
RAD51D				
RAD52				
RAD54				
RAF1				
RANBP2				
RANTES				
RAP1				
RAP1A				
RAP2C				
RAPTOR				
RB				
RB1				
RBP4				
RBP7				
RECQL4				
RELN				
REN				
RET				
RETN				
REV1				
REV3L				

RFC				
RGS21				
RHEB				
RHOA				
RIF1				
RIF2				
RIT1				
RMDN3				
RMI1				
RMI2				
RNF126				
RNF5				
RNF6				
ROCK				
RORC				
RPA				
RPA1				
RPA2				
RPA3				
RPA4				
RPTOR				
RRAGA				
RRAGB				
RRAGC				
RRAGD				
RRM2B				
RTEL1				
RTN4R				
RXRA				
RXRG				
S100A10				
S100B				
SAMD10				
SAMD9L				
SCARB1				
SCO1				
SCO2				
SDC1				
SDC2				
SDC3				
SDC4				
SDHA				
SDHAF1				
SDHB				
SDHC				

SDHD				
SEC61A1				
SELE				
SELENOT				
SELL				
SELP				
SEPINE1				
SEPTIN9				
SERPINA1				
SERPINA3				
SERPINA7				
SERPINC1				
SERPINE1				
SERPINF2				
SERPINI1				
SERT				
SFTPA				
SFTPA2				
SFTPB				
SFTPC				
SFTPD				
SHFM1				
SIRT1				
SIRT2				
SIRT3				
SIRT4				
SIRT5				
SIRT6				
SIRT7				
SKAP1				
SLC12A3				
SLC17A5				
SLC25A4				
SLC25A6				
SLC27A1				
SLC27A2				
SLC27A3				
SLC2A1				
SLC2A10				
SLC2A4				
SLC36A4				
SLC38A9				
SLC5A4				
SLC6A15				
SLC6A2				

SLC6A4				
SLC6A8				
SLC7A9				
SLX4				
SMAD				
SMAD4				
SMARCAL1				
SNAI1				
SNRPN				
SOAT1				
SOAT2				
SOD1				
SOD2				
SOD3				
SON				
SORL1				
SOS1				
SOX2				
SPP1				
SPRTN				
SQSTM1				
SRC				
SREBF1				
SREBP1				
SREBP1C				
SRL				
SST				
STAG2				
STAT3				
STK11				
STK32A				
STUB1				
SULT2A1				
SURF1				
SUZ12				
SZT2				
TAC1				
TACC3				
TANGO2				
TAS1R1				
TAS1R2				
TAS1R3				
TAS2R38				
TBC1D23				
TCAB1				

TCF7L1				
TCF7L2				
TDO2				
TERC				
TERF1				
TERF2				
TERF2IP				
TERRA				
TERT				
TET1				
TET2				
TET3				
TFAM				
TFEB				
TFIIH				
TGFA				
TGFB				
TGFB1				
TGFB2				
TGFB3				
TIMELESS				
TIMP1				
TIMP2				
TIMP3				
TIMP4				
TIN2				
TINF2				
TIPIN				
TJP1				
TJP2				
TJP3				
TLK1				
TLN2				
TLR2				
TLR3				
TLR4				
TLR7				
TLR8				
TM6SF2				
TMEM106A				
TMEM70				
TMPRSS2				
TNF				
TNFA				
TNNI3				

TNNT2				
TOMM20				
TOMM34				
TOP1				
TOP2A				
TOP2B				
TP53				
TP53BP1				
TP63				
TPH1				
TPH2				
TPS1				
TRAC1				
TRBC1				
TRDMT1				
TREM2				
TREML1				
TRF1				
TRF2				
TRIB3				
TRIM25				
TRIM27				
TRIM58				
TRIM59				
TRIP13				
TSC1				
TSC2				
TTC19				
TUBB2A				
TUBB3				
TWIST1				
TXA2				
TXN1				
TXN2				
TXNRD2				
UBC				
UBD				
UBE2A				
UBE2B				
UBE2D1				
UBE2D2				
UBE2E1				
UBE2N				
UBE2T				
UBR5				

UCHL5				
UCP1				
UCP2				
UCP3				
UCP4				
ULK1				
ULK2				
UPS				
USP1				
USP7				
UTS2				
VCAM1				
VCL				
VCP				
VDAC				
VDAC1				
VEGF				
VEGFA				
VEGFC				
VEGFD				
VEGFR				
VEGFR1				
VEGFR2				
VIM				
VIP				
VSIR				
VWF				
WDR24				
WFS1				
WNT1				
WNT3A				
WNT5A				
WNT7A				
WRAP53				
WRN				
XAB2				
XBP1				
XIST				
XPA				
XPC				
XPF				
XPG				
XRCC1				
XRCC2				
XRCC3				

XRCC5				
XRCC6				
ZBTB44				
ZBTB7A				
ZFR2				
ZMPSTE24				
ZMYND11				
ZNF335				
ZNF428				
ZNF488				
ZNF518B				
ZNF687				
ZNF827				
ZNF830				
ZWINT				
ZYG11A				
38261				
53BP1				
