# Supplementary figures and images for: Unravelling Convergent Signaling Mechanisms Underlying the Aging-Disease Nexus Using Computational Language Analysis
Source: Curr Issues Mol Biol. 2025 Mar 14;47(3):189. doi: 10.3390/cimb47030189 (PMC11941692; doi:10.3390/cimb47030189)

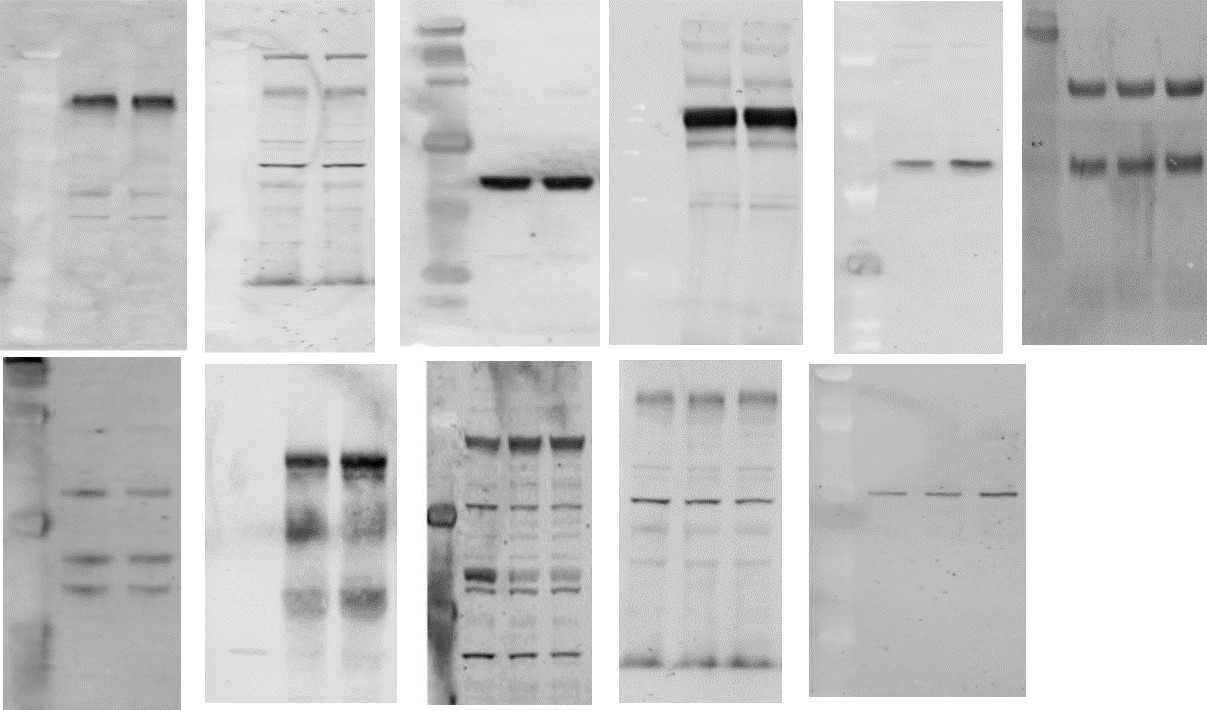

Supplement: Supplementary file 1 [file cimb-47-00189-s001.zip › Gel-Files.jpg]

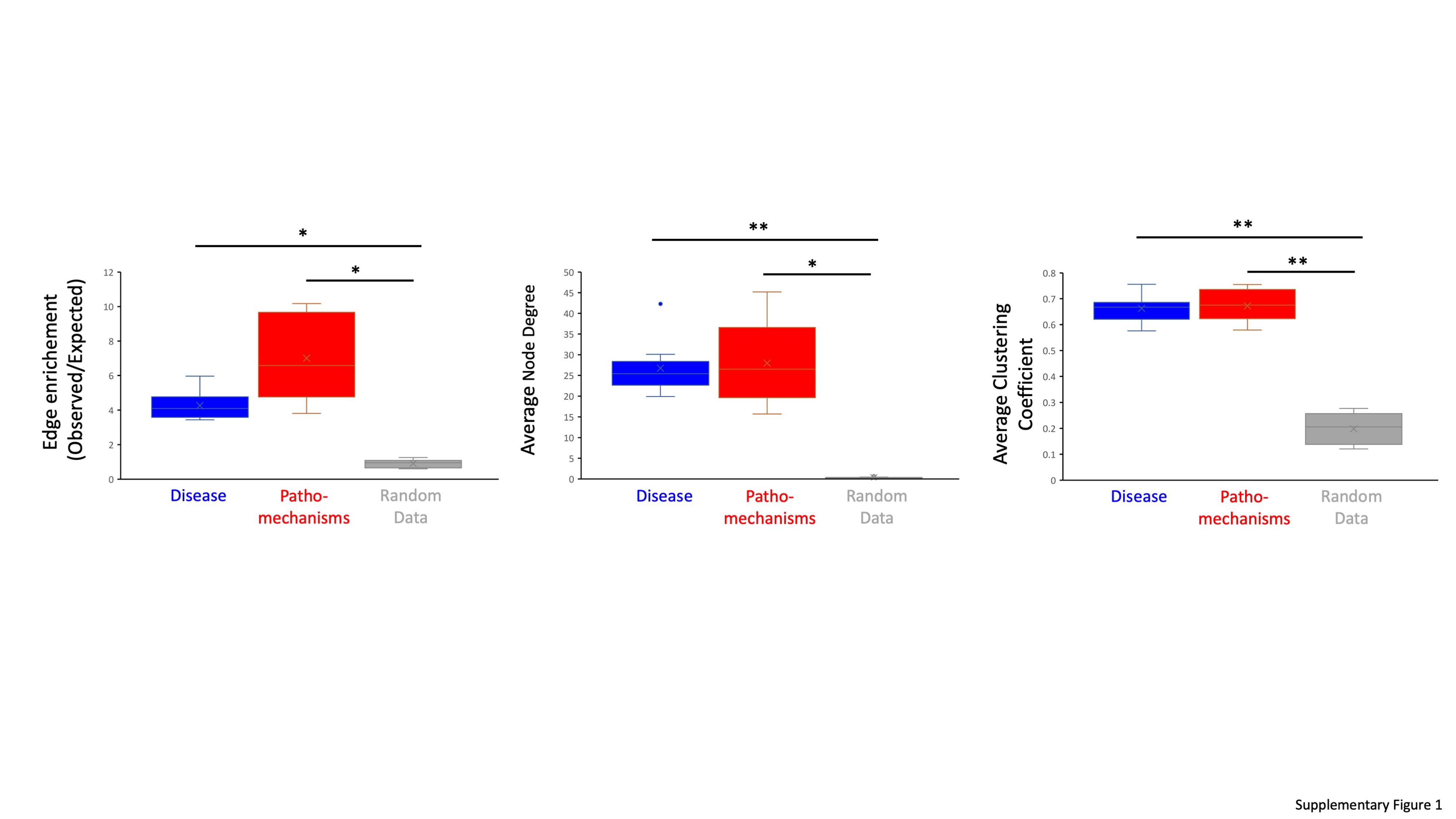

Supplement: Supplementary file 1 [file cimb-47-00189-s001.zip › CIMB-FigS1 .jpg]

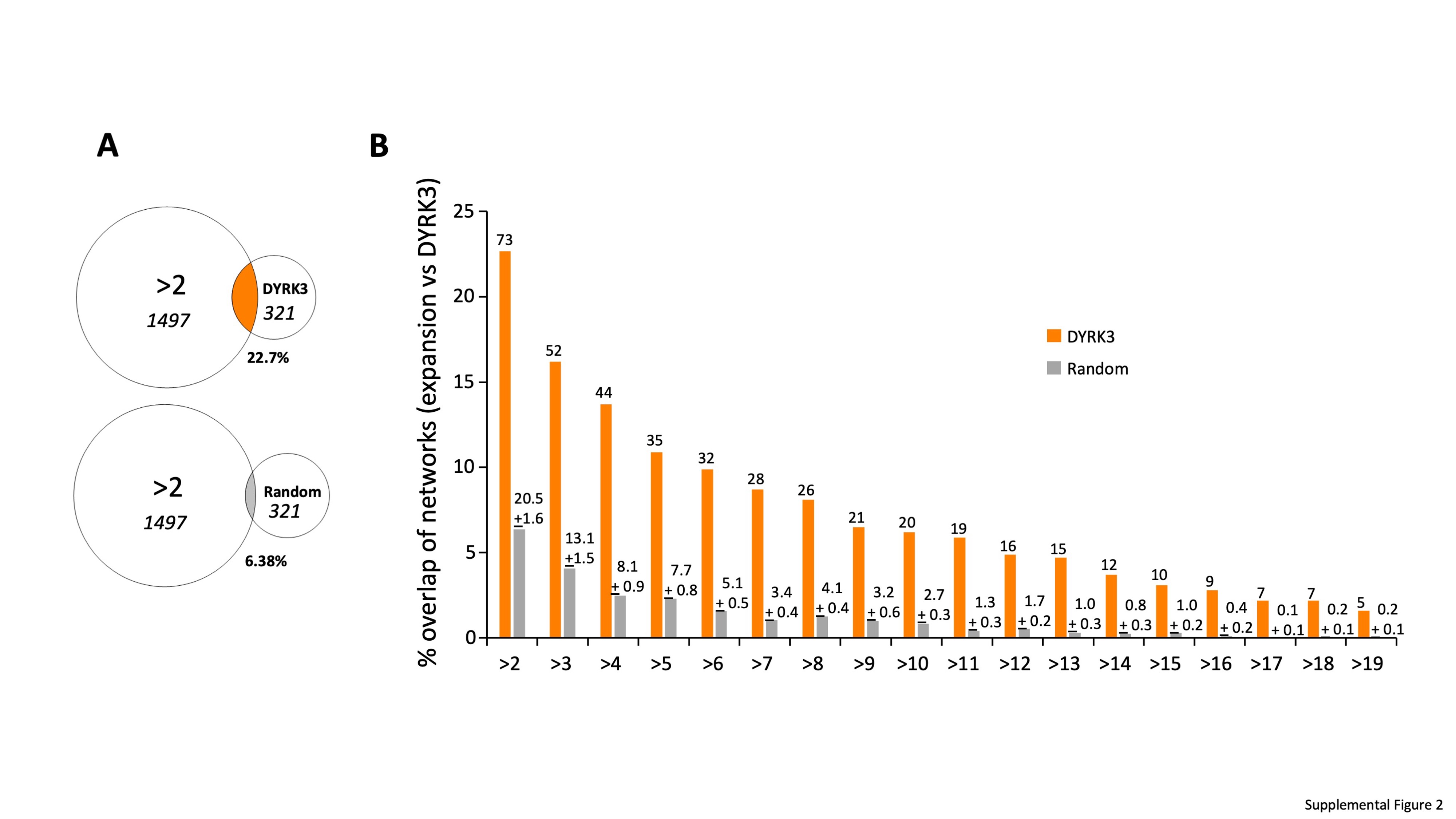

Supplement: Supplementary file 1 [file cimb-47-00189-s001.zip › CIMB-FigS2.jpg]

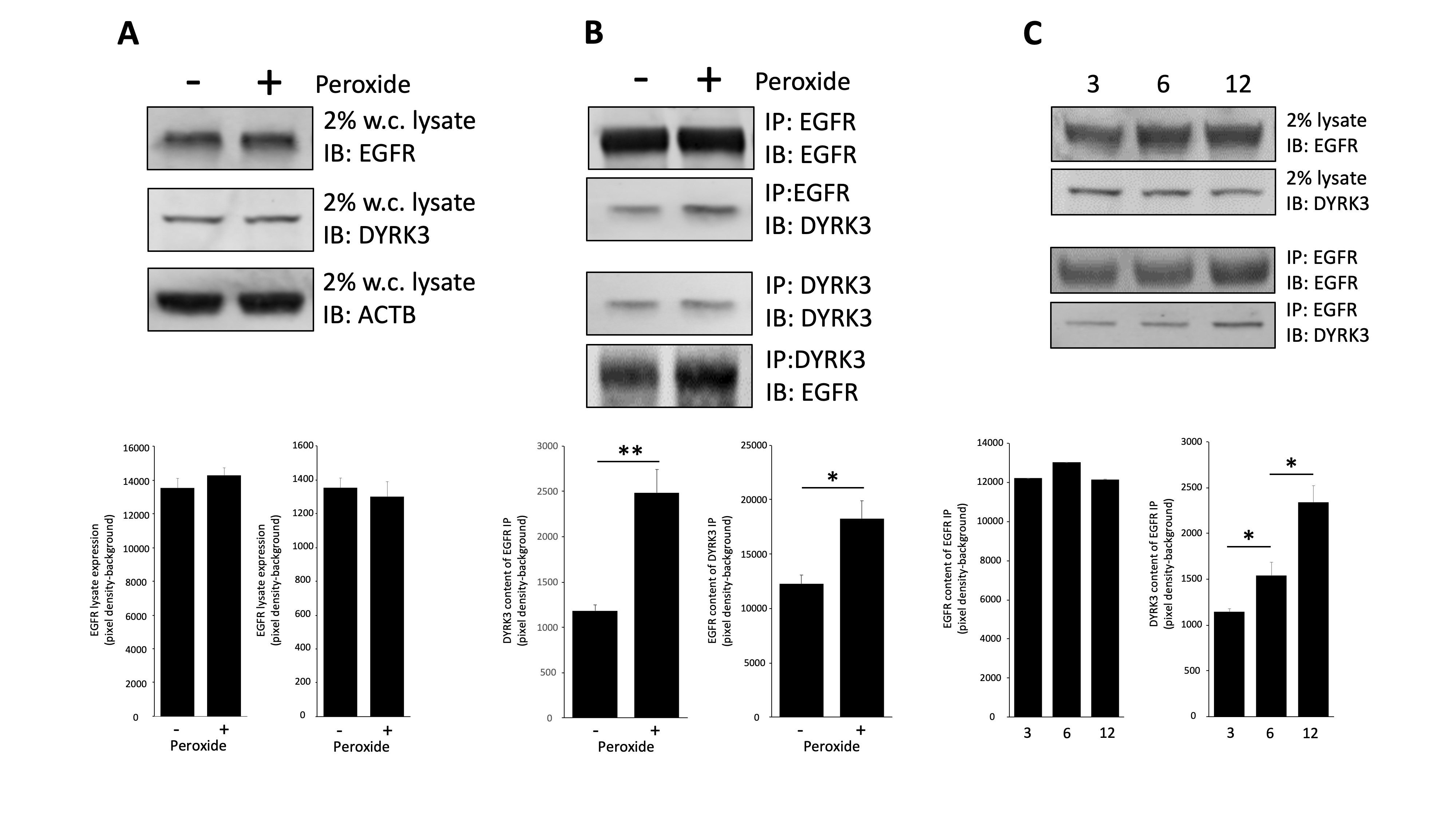

Supplement: Supplementary file 1 [file cimb-47-00189-s001.zip › CIMB-FigS3 .jpg]
